# Fluctuating salinity during development impacts fish life histories

**DOI:** 10.1111/1365-2656.70095

**Published:** 2025-07-01

**Authors:** Meng‐Han Joseph Chung, Daniel W. A. Noble, Rebecca J. Fox, Lauren M. Harrison, Michael D. Jennions

**Affiliations:** ^1^ Division of Ecology and Evolution Research School of Biology, Australian National University Acton Australian Capital Territory Australia; ^2^ Centre for Conservation Ecology and Genomics Institute for Applied Ecology, University of Canberra Bruce Australian Capital Territory Australia; ^3^ School of Biological Sciences University of East Anglia, Norwich Research Park Norwich UK

**Keywords:** environmental fluctuations, fast‐slow continuum, predictive adaptive response, salinity, silver spoon

## Abstract

1. Climate change and human activities are elevating the level and variability of salinity in freshwater ecosystems. Consequently, many aquatic species now experience more extreme developmental environments. Resultant shifts in developmental trajectories could change key life‐history traits that persist into adulthood.
2. The ‘silver spoon’ hypothesis posits that favourable developmental conditions lead to faster growth, earlier maturation and greater reproductive success. In contrast, the ‘predictable adaptive response’ hypothesis suggests that faster growth and earlier reproduction should be selected for under stressful developmental conditions because stress provides cues about a higher risk of mortality in future environments.
3. To understand life‐history responses to salinity during development, we reared a global pest, mosquitofish (*Gambusia holbrooki*), from birth in either freshwater control (0‰), stable‐saline (10‰), or fluctuating‐saline environments (0‰–20‰; mean = 10‰) until maturation. We then monitored their performance in early and late adulthood in a common garden setting.
4. Fish in fluctuating salinity grew more slowly and had a reduced reproductive output (lower sperm count, smaller eggs) than those in stable elevated salinity. These differences are consistent with a more stable environment providing a ‘silver spoon’ effect. Conversely, fish in stable elevated salinity grew faster and matured earlier than those in freshwater, supporting a ‘predictive adaptive response’ whereby salinity is a stressor triggering faster development and accelerates reproduction.
5. In addition, fluctuations in salinity altered the effect of higher salinity on self‐maintenance. Stable elevated salinity caused a decrease in male telomere length and female gut length, but fluctuating salinity caused an increase in female gut length.
6. Our results suggest that fluctuating versus stable salinity during development leads to distinct fish life histories. The effect sizes for some traits differed significantly between males and females, suggesting sex‐specific responses to climate fluctuations.

## Graphical abstract

Global freshwater ecosystems are increasingly threatened by salinisation. Salinity levels not only rise but also fluctuate due to extreme climate events and human activities. This study shows that stable elevated salinity accelerates reproduction in a freshwater fish, but fluctuations in salinity reduce reproductive output, with sex‐specific effects on these changes.

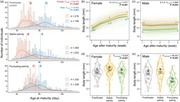

## 摘要

1. 氣候變遷與人類活動正提高淡水生態系中的鹽度濃度與波動程度，導致許多水生動物在發育階段面臨更極端的環境。這些變化可能改變個體的發育軌跡，而後影響成體階段的性狀表現。
2. 「銀湯匙」假說主張，穩定良好的發育環境可促進體型成長、加速性成熟、提高繁殖成功。相反地，「可預期適應反應」假說認為，個體會在有壓力的發育環境下加速體型成長、提早性成熟，因為這些個體預測未來的高死亡風險。
3. 本研究探討在發育階段的鹽度變化會對淡水魚的成長、繁殖與身體機能造成什麼影響。我們將剛出生的大肚魚 (*Gambusia holbrooki*) 分別飼養於淡水對照組、穩定鹽度組、波動鹽度組直到性成熟。而後將所有成體飼養在淡水中，並追蹤公母魚在成體早晚年的性狀表現。
4. 發育在穩定鹽度組的個體成長較快、繁殖產出高於波動鹽度組(包含較多的精子、較大的魚卵)，符合「銀湯匙」假說:即穩定的環境可促使個體發育與繁殖。相反地，淡水對照組的魚隻成長較慢、性成熟時間晚於穩定鹽度組，符合「可預期適應反應」假說:即鹽度作為一種壓力因子，可促使個體加速發育與繁殖。
5. 鹽度波動也改變高鹽濃度對身體機能的影響。在穩定高鹽度下，雄魚的端粒長度與雌魚的腸道長度皆減少；但在波動鹽度下，雌魚的腸道長度增加、雄魚端粒長度不變。
6. 本研究發現發育環境中的鹽度與其波動程度可顯著改變魚類生活史，而且此改變具有性別專一性。

## 1 INTRODUCTION

Climate change is now a major driver of extinction (Dawson et al., 2011), partially because populations are experiencing more variable environmental conditions (Arnell et al., 2019; Thornton et al., 2014). Understanding how environmental variability affects life histories is key to predicting population viability in a changing climate. Rapidly fluctuating environments create shorter periods of more intense stress than are typical in stable environments, implying that variability is harmful (Nielsen et al., 2003). However, greater variability also involves periods of reduced stress that might promote recovery (Rilov et al., 2019). Natural selection favours life‐history strategies that optimise allocation to growth, reproduction and self‐maintenance, enabling organisms to persist in diverse habitats (Stearns, 1992). In stable environments, selection is expected to promote local adaptation of life history (Wiersma et al., 2007). Optimal life‐history strategies partly depend on how environmental factors affect extrinsic rates of mortality at different life stages (de Vries et al., 2023), and how shifts in resource allocation moderate the risk of mortality (Healy et al., 2019).

**FIGURE 1:**
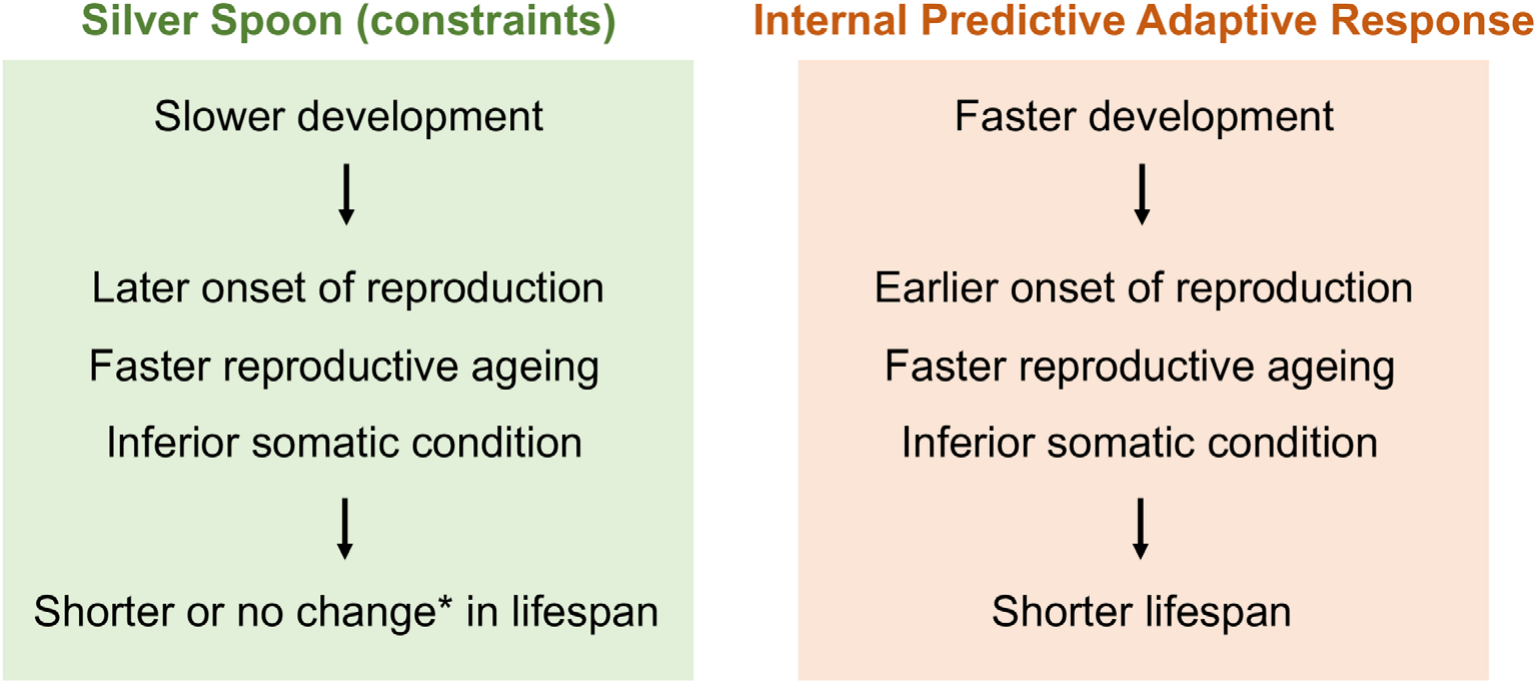
Predictions demonstrating the effect of developmental stress on life‐history trajectories. *See meta‐analysis: Cooper and Kruuk (2018).

Juveniles are often more vulnerable to environmental stressors than adults (Alderdice, 1988; Byrne, 2011). Ontogeny can irreversibly affect adults (Fischer et al., 2014; Griffin et al., 2018), and several hypotheses have been proposed about the life‐history consequences of developmental stress. The ‘silver spoon’ hypothesis (Grafen, 1988) posits that favourable environments during development enhance growth, accelerate maturation and produce more reproductively successful adults (Douhard et al., 2014; Lea et al., 2015; Madsen & Shine, 2000; Manzano Nieves et al., 2019)—such as females producing more eggs (Sanghvi et al., 2021) or males being more sexually attractive (Kahn et al., 2012)—plausibly due to lower expenditure on somatic maintenance for survival (Monaghan, 2008). In contrast, the ‘predictive adaptive response (PAR)’ hypothesis predicts that stress during development will accelerate growth and hasten maturation (Berghänel et al., 2017; Dantzer et al., 2013) because stress provides cues about higher rates of mortality that select for earlier reproduction (Belsky et al., 2015; Ellis et al., 2009). Adaptive responses require the ability to detect and act upon reliable cues about future environments (i.e. ‘external’ PAR; Bateson et al., 2004; Gluckman et al., 2005), but this capability is less likely in novel environments (Ghalambor et al., 2007). It is possible, however, that, even if environmental cues are undetected, somatic damage directly imposed by a novel environment could signal the risk of mortality and promote an earlier onset of reproduction (i.e. ‘internal’ PAR; Berghänel et al., 2016; Nettle et al., 2013). These varying predictions (Figure 1) highlight the difficulty of predicting life‐history responses to novel developmental stress.

Males and females usually experience sex‐specific selection, so their life‐history responses to developmental stress may differ. Females in many taxa (e.g. fish, reptile) tend to benefit from ongoing growth because fecundity increases with body size (Armstrong et al., 2018; Barneche et al., 2018). Conversely, males often allocate more heavily to sexually selected traits (e.g. courtship) than somatic maintenance, which is associated with faster senescence (Clutton‐Brock & Isvaran, 2007) and greater mortality (Kraus et al., 2008). Consequently, females are often more stress resistant than males (Bonduriansky et al., 2008; Mauvais‐Jarvis, 2024). But, despite these sex differences, we often lack suitable data from males in life‐history studies (Archer et al., 2022; Lemaître et al., 2015; but see Chung et al., 2024; Riesch et al., 2016).

Here, we investigate both male and female life‐history responses by a freshwater fish to fluctuating versus stable environmental stress during development. For aquatic species, climate change is not only elevating water temperatures but also changing salinity (Cunillera‐Montcusí et al., 2022). Freshwater fish osmoregulate to combat water loss and ion gain when salinity increases; and marine fish do the opposite when salinity decreases (Evans, 2008). These processes are energy‐demanding (Tseng & Hwang, 2008). Except for a few euryhaline species, most fish cannot readily switch their mode of osmoregulation (Kültz, 2015), implying that fluctuations in salinity are harmful (Nielsen et al., 2003). Salinity fluctuations occur naturally due to variation in precipitation and seawater intrusion into freshwater, but human activities amplify these fluctuations by altering water run‐off (e.g. irrigation) and adding salt to freshwater (e.g. industrial effluents) (Cañedo‐Argüelles et al., 2019; Mirzavand et al., 2020). Currently, salinisation affects over 30% of freshwater ecosystems (Iglesias, 2020). For example, higher salinity in the Aral Sea shifted the ecosystem from freshwater to marine, collapsing local fisheries (Aladin et al., 2009). Many studies have asked how marine or estuarine populations acclimate to lower salinity (e.g. Islam et al., 2024; Mozanzadeh et al., 2021; Yoshida & Tomiyama, 2021). However, of 193 recent studies on salinisation (review: Cunillera‐Montcusí et al., 2022), few (if any) have investigated how *fluctuating* salinity impacts life histories of freshwater fish (also see: Lee et al., 2022).

In this study, we reared newborn mosquitofish (*Gambusia holbrooki*) in freshwater versus constant or fluctuating saltwater. To evaluate predictions about life‐history trajectories (Figure 1), we measured a suite of traits (growth, reproduction, survival, self‐maintenance) in early and late adulthood for both sexes. Mosquitofish are among the most invasive freshwater fish globally (Pyke, 2008). They are hard to eradicate, partly because of their tolerance of environmental stressors (Pyke, 2008). However, established populations have ready access to saline waters arising from the drying of streams and salinisation after land clearing (Brown‐Peterson & Peterson, 1990; Morgan et al., 2003; Tumlison, 2017). In saline water, mosquitofish are less aggressive (Alcaraz et al., 2008), take longer to explore novel environments (Zhou et al., 2022) and have increased costs of osmoregulation (Tsai et al., 2018). These observations are, however, all based on short‐term studies of adults. The life‐history response to prolonged exposure to salinity throughout development remains unknown.

Physiological responses to acute salinity changes are costly: fish must rapidly increase emergency energy use (e.g. glucose) in the brain, liver, kidney and gill tissues to initiate active ion transports, as well as reverse osmoregulatory modes in the opposite direction (e.g. from ion absorption in freshwater to ion secretion in saltwater) (Evans & Kültz, 2020). Costly energy expenditures may decline after long‐term acclimation to a stable‐saline environment. However, in a fluctuating‐saline environment, energetically costly changes in osmoregulatory physiology occur repeatedly. Given there are higher energetic costs in fluctuating environments, we predict that (I) fluctuations in salinity will magnify any life‐history responses induced by stable elevated salinity. In other words, both fluctuating‐ and stable‐saline environments will cause similar changes in trait performance relative to freshwater, but the magnitude of these changes will be greater in the fluctuating environment.

Reproductive success typically increases with the number of breeding attempts. Unlike *Gambusia* males that can mate again within minutes (Wilson, 2005), females have a longer ‘time out’ period after fertilising eggs (Koya et al., 1998), meaning that longevity has a greater influence on the total number of broods they can produce. *Gambusia* females also have greater tolerance than males to environmental stress (e.g. low temperature, starvation) (Li et al., 2017; Wood et al., 2020). We therefore predict that (II) females that experience elevated salinity will develop more slowly and delay the onset of reproduction to repair salinity‐induced damage (which is expected to be less severe than in males), thereby increasing their chances of adult survival (i.e. freshwater creates a ‘silver spoon’ effect). In contrast, we predict that (III) males that experience elevated salinity will show an earlier onset of reproduction, faster senescence and a shorter lifespan (i.e. a ‘predictive adaptive response’ in both saline environments). This is because males often express sexually selected traits that compromise somatic maintenance (Zuk, 2009) and/or engage in risky mating behaviours that increase mortality (Emery Thompson & Georgiev, 2014). Indeed, in freshwater, *G. holbrooki* males show minimal adult growth, allocate heavily to trying to mate and senesce and die sooner than females (Chung et al., 2024; Pyke, 2005). Stronger sexual selection on males than females favours investment in male traits that maximise reproductive success at the expense of survival (Janicke et al., 2016), which aligns well with the expectation of a ‘predictive adaptive response’: accelerated reproduction in anticipation of higher future mortality.

## 2 METHODS

### 2.1 Origin and maintenance of fish

We collected mosquitofish from a stream (35°180′27′′ S 149°070′27.9′′ E) in Canberra, Australia, in 2020–2021. Wild‐caught juveniles were reared in 90 L mixed‐sex stock aquaria filled with freshwater until they could be sexed (males have a pointed anal fin) and transferred to single‐sex aquaria to ensure their virginity.

Wild‐caught adults were housed in 90 L mixed‐sex stock freshwater tanks. Pregnant females were transferred to individual 1 L tanks to give birth (*n* = 118 broods). We randomly assigned newborn fry (i.e. focal fish) from each brood across three treatments (maximum 11 fry per brood/treatment): (a) freshwater (*n* = 406), (b) stable elevated salinity (*n* = 487) or (c) fluctuating salinity (*n* = 467). Siblings assigned to the same treatment were housed in communal tanks (at <1 individual per litre), at the same density for each treatment for each brood. The three tanks for each brood (one per treatment) were placed close together in a randomised order in the same temperature‐controlled room. We accounted for any potential differences among broods in all analyses by including brood identity as a random factor (see below).

All fish were kept under a 14L:10D cycle at 28 ± 1°C and fed twice daily on commercial fish flake and *Artemia* nauplii (for stock fish) or *Artemia* nauplii only (for focal fish).

### 2.2 Experimental design

The richness of freshwater species declines dramatically at ≥10‰ salinity (Pinder et al., 2005; Telesh & Khlebovich, 2010), a threshold now being exceeded in many freshwater bodies (Beatty et al., 2011; Nielsen et al., 2003; Vidal et al., 2021). We kept juveniles in the *stable*‐*saline* treatment at a constant 10‰. Fish need up to 3 days to switch osmoregulatory modes (Evans & Kültz, 2020), so we reared juveniles in the *fluctuating‐saline* treatment at salinities that cyclically changed every 2 days (10‰ → 20‰ → 10‰ → 0‰ → 10‰) with the same mean level (10‰). We initially transferred newborn fry assigned to the *stable* and *fluctuating* treatments into tanks with 2.5‰ saline water for 2 days, while juveniles assigned to the *freshwater* treatment were always kept in 0‰ water. No initial mortality occurred (personal observation). Fluctuating‐salinity levels were created by rotating fish between tanks with different salinity concentrations. Juveniles in the *freshwater* and *stable‐saline* treatments were also rotated among tanks of 0‰ or 10‰ salinity, respectively, to control for any effects of changing tanks. To generate saline water, we dissolved aquarium salt (Aquaforest, Brzesko, Poland) in tap water and aquatic conditioner (Seachem, Madison, USA). We checked the salinity level every 2 days using a refractometer and renewed the water weekly.

We inspected tanks every 2 days to check for sexual maturity and recorded the age of maturity. To investigate the effect of developmental environment on adults, we transferred newly matured focal fish into individual 1 L freshwater tanks (i.e. common garden setting) for 1 week before measuring their standard length (SL: the snout tip to the end of the vertebral column). We estimated life‐history trajectories by quantifying adult performance when:

- *Young*: One week post‐maturation, virgin females (*n* = 290) and males (*n* = 240) (at least one per sex/brood/treatment) were randomly euthanised to measure life‐history traits (see below).
- *Old*: We introduced the remaining females (*n* = 316) and males (*n* = 327) into 4 L freshwater mating tanks, each with a stock fish of the opposite sex for 12 weeks (~75% of the breeding season length in the source population; Kahn et al., 2013). Focal adults were free to mate with the stock fish. Before being placed into mating tanks, focal male mating behavior and sperm traits were measured (see below). The stock fish were rotated fortnightly between tanks to maintain the sexual interest of the focal fish. Any deaths were recorded. Subsequently, we measured the same life‐history traits of the surviving females (*n* = 284 of 316; 90%) and males (*n* = 277 of 327; 85%).

#### 2.2.1 Body length

Focal fish were anaesthetised in ice‐cold water and placed on a glass slide. The side of their body was photographed to measure their SL using *ImageJ*.

#### 2.2.2 Growth

We placed a juvenile (maximum five per brood/treatment; *n* = 888) in a shallow container of tank water and photographed it from above every fortnight (0, 2, 4 and 6 weeks after birth) to determine its SL. We followed the same procedure to measure adult growth during the mating period (0, 2, 4, 6 and 8 weeks).

#### 2.2.3 Male mating behaviour

We placed each male into a 7 L tank with a virgin female (SL: 29.8 ± 0.09 mm; *n* = 559) separated by a mesh screen. After 10 min, we removed the screen and recorded the following male behaviors for 20 min: (a) number of mating attempts (gonopodium swings forward under the female's gonopore), (b) number of successful mating attempts (gonopodium contacts the gonopore), (c) time spent with the female (distance <1 SL from the female).

#### 2.2.4 Sperm traits

After the behavioural trial, we measured total sperm count and velocity following methods in Vega‐Trejo et al. (2019). Briefly, we pressed a male's abdomen to empty his sperm reserves. After 7 days, we repeated this process to obtain new sperm with a standardised age. Sperm was collected with extender medium for counting. For sperm velocity, we collected two separate samples of four sperm bundles with extender medium. Three microlitres of each sample was placed in a multi‐test slide (MP Biomedicals, USA) and activated using 125 mM KCl and 2 mg/mL bovine serum albumin. Sperm traits were measured using a sperm tracker (Hamilton Thorne Research, USA) (Supporting Information).

#### 2.2.5 Female fecundity

After immunity measurement (see below), females were euthanised to measure their fecundity. For young females (virgins), we counted and photographed their mature eggs and measured their diameter using *ImageJ*. For old females (after the 12‐week mating period), we counted the number of: (a) broods (range: 0–2) and (b) offspring born during the mating period; (c) embryos (i.e. fertilised eggs) and (d) unfertilised eggs. We defined (b) + (c) as the total number of offspring; and (c) + (d) as the total number of eggs.

#### 2.2.6 Immune response

Long‐term stress is often associated with immunosuppression in fish (Wendelaar Bonga, 1997). We used a phytohemagglutinin injection assay to quantify cell‐mediated immunity (Iglesias‐Carrasco et al., 2019). When fish were anaesthetised, we recorded the thickness of their body at the posterior end of the dorsal fin five times with a pressure‐sensitive spessimeter (Mitutoyo 547‐301; accuracy: 0.01 mm). At the same location, we then injected 0.01 mg of phytohemagglutinin dissolved in 0.01 mL of PBS into the left side of the fish. After 24 h, the body thickness was again measured five times (repeatability: *r* ± SE = 0.992 ± 0.000, *p* < 0.001, *n* = 1114 measure‐female‐age; 0.978 ± 0.001, *p* < 0.001, *n* = 1022 measure‐male‐age). We treated the difference in mean pre‐ and post‐injection thickness as an index of immunity.

#### 2.2.7 Gut length

The gut aids salinity acclimation through ion uptake that reduces gut osmolarity to facilitate water uptake (Grosell, 2006). However, intestinal cells have a high turnover rate (Cant et al., 1996), suggesting that having a longer gut is costly (Aiello & Wheeler, 1995; Kotrschal et al., 2013). Fish were fed in the same way across all three treatments to minimise diet‐induced changes in gut length. At the end of the experiment, we euthanised fish using *Aqui‐S* (0.0075% v/v) after the immunity assay. Their guts (the digestive tract from mouth to anus) were dissected under a microscope, photographed and measured with *ImageJ*. We predicted a longer gut length in both stable‐ and fluctuating‐saline environments due to increased demand for osmoregulation.

#### 2.2.8 Relative telomere length

Telomere shortening is often linked to ageing (López‐Otín et al., 2013) and death (Wilbourn et al., 2018). To measure relative telomere length (RTL), we randomly selected 30 fish per sex/age/treatment (total *n* = 360) and collected their tail muscle (20 mg). We used real‐time qPCR to measure RTL by comparing the ratio (T/S) of telomere repeat copy number (T) to a single‐copy gene copy number (S) against a reference DNA sample (see Supporting Information).

### 2.3 Statistical analysis

Analyses were run using R v4.0.5. We used the *Anova* function of the *car* package (type III Wald *chi‐square* or *F*‐tests with Kenward‐Roger *df*) to determine *p* values. Summary statistics are mean ± SE. We first examined the results with *α* = 0.05 (two‐tailed) and then applied a Bonferroni correction (see Table S1) to assess whether any significant effects might be false positives.

To test the effect of developmental environment on measured traits and whether it was age‐dependent, we ran initial models with environment (freshwater, stable‐saline, fluctuating‐saline), age (young, old) and their interaction as fixed factors. Age was excluded if a trait was only measured at one age (e.g. egg size). If there was a significant interaction, we examined the environmental effect separately at each age. If the interaction was non‐significant, it was removed from the model to estimate the main effects of environment and age (Engqvist, 2005). The best fit of the model remained unchanged after excluding the non‐significant interaction (Tables S5 and S6). Given a significant environment effect, we ran post hoc Tukey's tests to test for pairwise differences. For pairwise comparisons between treatments, we retained *α* = 0.05 as Tukey's HSD tests account for multiple testing (Nanda et al., 2021). Each trait was analysed with a separate model. All models were run separately for each sex given there are well‐known sex differences in life‐history responses (Kahn et al., 2013; Vega‐Trejo et al., 2018). Brood ID was included in all models as a random factor using (1 + age | brood ID) to account for any potential heritable differences and/or variation in rearing conditions among broods, with respect to mean values and the effect of age (i.e. allowing the effect of being ‘young’ or ‘old’ to vary across broods). We included individual ID as a random factor for male reproductive traits to account for repeated measures from the same males at both ages.

For all traits, we first ran linear mixed models (LMMs) and checked whether the model residuals fulfilled the assumptions of normality, homoscedascity and linearity via Q–Q plots. If not, we either log‐transformed the data (sperm count, gut length) or ran generalised linear mixed models (GLMMs) with an appropriate error distribution: (a) quasi‐Poisson error for total egg number, (b) negative binomial error for total egg number of young females and (c) negative binomial error with zero‐inflation for the number of mating attempts, total number of offspring and number of embryos. We included the same variables in zero‐inflated models as those in main models. For traits that contained mostly 0 and 1 responses, we set the >1 data to 1 and ran models using (d) binomial error, including male successful copulation (only 4% of the males copulated successfully more than once) and female brood number (only 2% of females had more than a brood). Due to convergence issue, we used (1| brood ID) instead of (1 + age | brood ID) when testing the environment‐by‐age interaction in the initial models for number of mating attempts. We removed (1| brood ID) in the zero‐inflated model of the number of mating attempts by old males. We ran overdispersion tests using the *DHARMa* package to ensure that the variance was not greater than the mean for GLMMs.

For immunity, we included standardised SL as a covariate to account for differences in swelling responses between larger and smaller individuals after the PHA‐injection challenge. For log‐transformed gut length, we accounted for its allometric relationship with body size (Figure S1) by log‐transforming SL and including it as a covariate (standardised) (Nakagawa et al., 2017). For RTL, we ran an additional LMM including age since birth (standardised) as a covariate to test how chronological age affects telomere shortening.

#### 2.3.1 Growth

We ran separate LMMs for growth in juveniles, adult females and adult males. We considered SL as the response variable, and treated environment as a fixed factor, and age (in weeks) as a fixed covariate. We treated brood ID (for both juveniles and adults) and individual ID (for adults) as random factors to control for repeated measures from the same family and/or fish. We included the environment‐by‐age interaction, and age^2^ to account for non‐linear growth. If the interaction was significant, we ran individual LMMs (and Tukey's tests) at each age to determine when body size diverged between environments.

#### 2.3.2 Mortality

Adult mortality was evaluated separately for each sex using Cox regression models (*coxme* package) with environment as the fixed factor and brood ID as a random factor. Surviving fish were treated as right‐censored data.

#### 2.3.3 Sex differences

We calculated the standardised mean difference (effect size = Cohen's *d*) for the effect of salinity (*stable saline* relative to *freshwater*) and the effect of fluctuations (*fluctuating* relative to *stable saline*) for traits in both sexes. We further tested the difference in effect sizes between females and males using Welch's *t*‐test (*α* = 0.05, two‐tailed) to assess sex‐specific responses.

## 3 RESULTS

**FIGURE 2:**
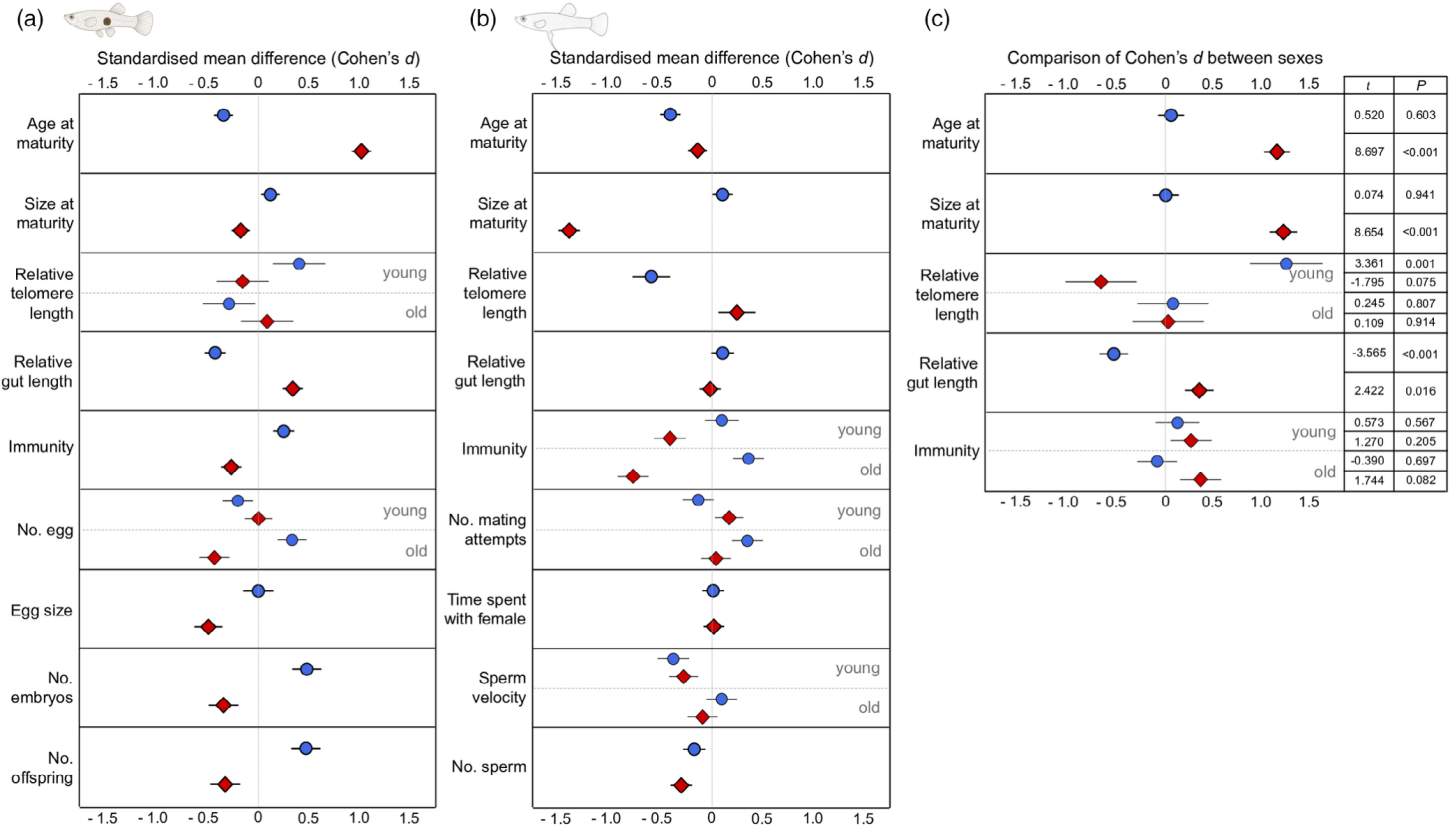
Standardised mean differences (Cohen's *d*) for traits in (a) females and (b) males, and (c) the comparison of effect size for traits jointly measured in both sexes. Blue circle = effect of salinity (*stable‐saline water* relative to *freshwater*). Red diamond = effect of fluctuations (*fluctuating‐saline* relative to *stable‐saline water*). Comparison of effect size represents the difference in Cohen's *d* in *females* relative to *males*. Effects are presented separately at each age class if there is a significant environment‐by‐age interaction (Table S1). Error bars represent mean ± SE (see Noble et al., 2017 for calculation). Welch's *t*‐tests (*α* = 0.05, two‐tailed) were used to test the significance of effect size comparisons. Figures only present conditional parts of the results for number of embryos, number of offspring and number of mating attempts.

The developmental environment had a significant effect on 22 of the 28 measured traits (Table S1), either through the main effect (on 12 traits) or its interaction with age (on 10 traits). After accounting for multiple testing, the environmental effects remained significant for 11 of the 28 traits (Table S1), far exceeding the rate expected by chance alone (2.8 of 28 traits, if *α* = 0.05 for the main effect or an interaction with age). Life‐history traits of males and females had a similar propensity to be affected by the environment (females: 4 of 14 traits; males: 6 of 13 traits; significant after Bonferroni correction), indicating that both sexes were sensitive to salinity during their development. To facilitate an accessible summary of the results, we provided a summary table (Table 1) and figures illustrating the standardised mean difference (Cohen's *d*) and the contrast in effect sizes between females and males for sex‐specific responses (Figure 2).

**TABLE 1:** Summary of the effect of salinity (*stable‐saline water* relative to *freshwater*) and the effect of fluctuations (*fluctuating‐saline* relative to *stable‐saline water*) on life‐history components (G = growth/development, S = self‐maintenance, R = reproduction).

Trait		Effect of salinity		Effect of fluctuations	
G	Juvenile growth	**+**		**−**	
Young adult stage		Female	Male	Female	Male
Age at maturity		**−** (younger)	**−** (younger)	**+** (older)	**×**
Size at maturity		+*	**×**	**×**	**−**
S	Relative telomere length	**×**	**−**	**×**	**×**
	Relative gut length	**−**	**×**	**+**	**×**
	Immunity	**×**	**×**	**×**	**×**
R	No. of eggs	**×**	NA	**×**	NA
	Egg size	**×**	NA	**−**	NA
	No. of mating attempts: zero‐inflation	NA	**×**	NA	**×**
	No. of mating attempts: condition	NA	**×**	NA	**×**
	Likelihood of successful mating	NA	**×**	NA	**×**
	Time pursuing female	NA	**×**	NA	**×**
	Sperm velocity	NA	**×**	NA	**×**
	No. of sperm	NA	**×**	NA	**−**
Old adult stage		Female	Male	Female	Male
S	Relative telomere length	**×**	**−**	**×**	**×**
	Relative gut length	**−**	**×**	**+**	**×**
	Immunity	**×**	+*	**×**	−*
R	No. of eggs	**×**	NA	−*	NA
	No. of embryos: zero‐inflation	−*	NA	**×**	NA
	No of embryos: condition	**×**	NA	−*	NA
	No. of mating attempts: zero‐inflation	NA	**×**	NA	**×**
	No of mating attempts: condition	NA	+*	NA	**×**
	Likelihood of successful mating	NA	**×**	NA	**×**
	Time pursuing female	NA	**×**	NA	**×**
	Sperm velocity	NA	**×**	NA	**×**
	No. of sperm	NA	**×**	NA	**−**
Throughout adulthood		Female	Male	Female	Male
Mortality		**×**	**×**	**×**	**×**
G	Adult growth	**+**	**×**	**−**	**−**
R	Likelihood of giving birth	**×**	NA	**×**	NA
	No. of offspring: zero‐inflation	**×**	NA	**×**	NA
	No. of offspring: condition	+*	NA	**×**	NA

*Note*: Significant effects were based on Tukey's tests for pairwise comparisons. ‘+/yellow’ indicates an increase in trait values, ‘−/blue’ a decrease, ‘×’ no significant effect, ‘NA’ unavailable data, and ‘*’ indicates an effect that became non‐significant after correcting for multiple testing.

Below we provide detailed results of environmental effects on trait performance in each sex.

### 3.1 Juvenile growth

**FIGURE 3:**
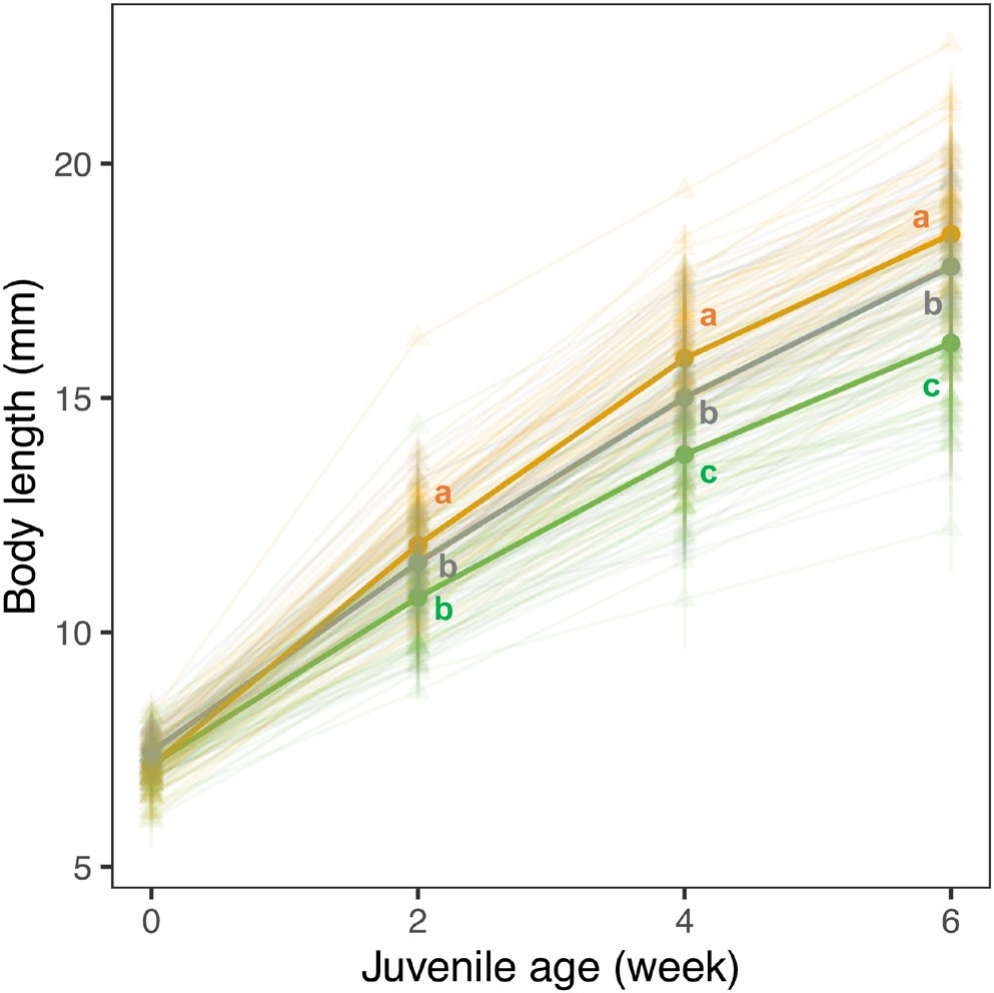
Environmental effects on juvenile growth. Colours indicate environments: Freshwater (grey); stable‐saline (orange); fluctuating‐saline (green). Juvenile size is shown at the brood level (triangle, thin line) along with the mean for each environment (circle, thick line). Different letters represent significant differences between environments from Tukey's tests. Line bars represent mean ± SE.

The developmental environment affected juvenile growth, but the effect depended on age (LMM, *F* = 220.088; *p* < 0.001; Figure 3). Except at birth (week 0), juveniles were consistently larger in the stable‐saline than in the other two environments (Tukey's tests: all *p* < 0.001). Juvenile growth did not differ between the freshwater and fluctuating‐saline environments during the first 2 weeks (Tukey's test: *p* = 0.887), but fish in the fluctuating‐saline environment were the smallest from week 4 onward (Tukey's tests: all *p* < 0.001; Table S2).

### 3.2 Age at maturity, size at maturity and adult growth

#### 3.2.1 Females

**FIGURE 4:**
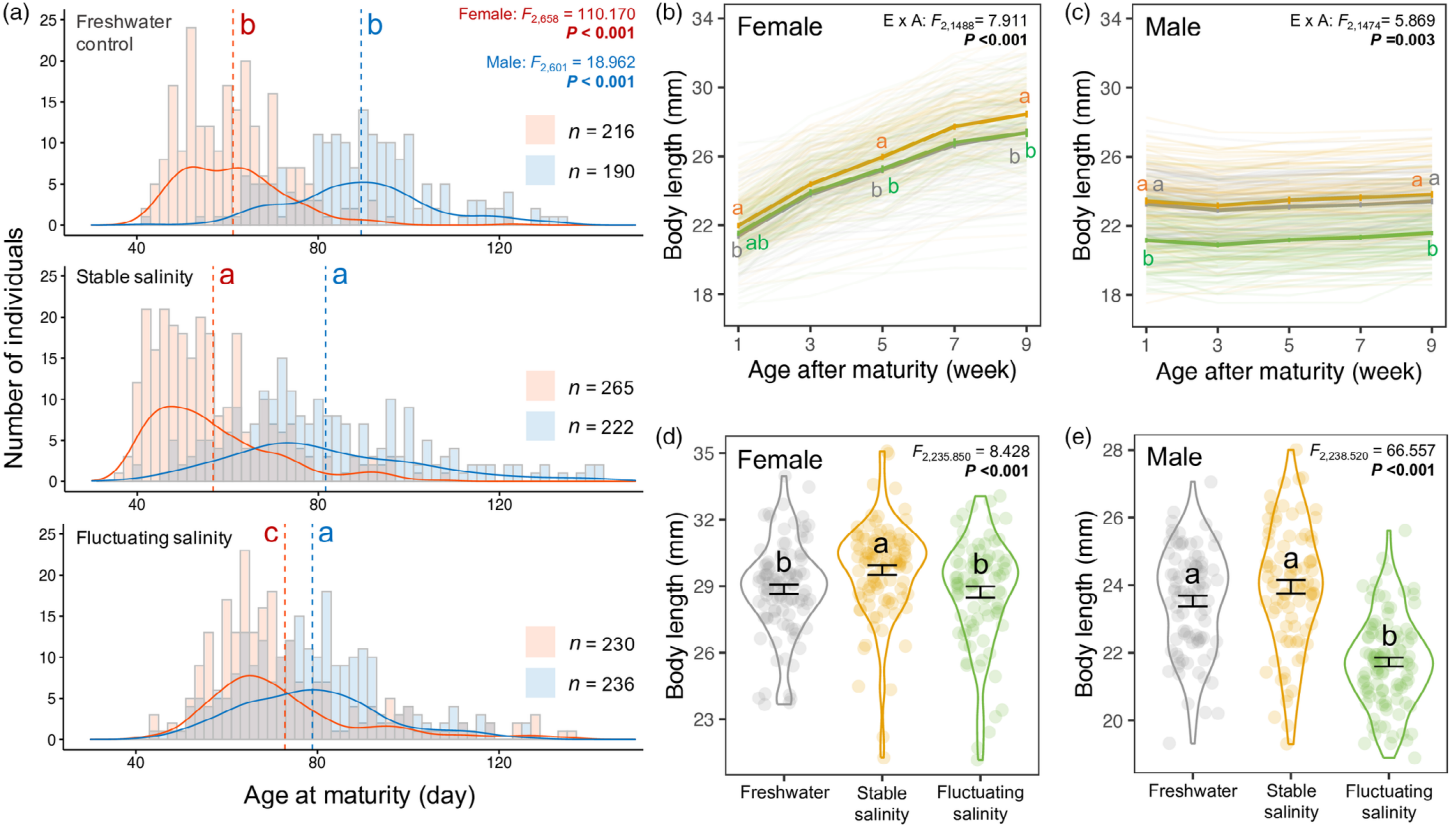
Environmental effects on (a) age at maturity, (b, c) adult growth and (d, e) body size after the breeding period for both sexes. Days to mature (a) of females (red) and males (blue) are shown individually for each environment in a combination of histogram and density plots (mean = dashed line). Line plots (b, c) show individual adult growth (thin lines) and mean growth (thick lines) in each environment in the first 8 weeks after returning to freshwater. Violin plots (d, e) show the body length at the end of the mating period (Week 13 following maturation for females; Week 15 for males). Colours represent three environments: Freshwater (grey); stable‐saline (orange); fluctuating‐saline (green). E × A indicates an environment‐by‐age interaction. Different letters represent significant differences among environments from Tukey's tests. For adult growth (b, c), letters are only shown at the start and end of the adult period and when the significance levels changed; no letter at an age indicates that the significant difference seen at the previous age persists (see Table S2). Error bars represent mean ± SE.

The developmental environment significantly affected female age and size at maturity (LMM, both *F* > 4.703; *p* < 0.010). Females matured later in the fluctuating than stable‐saline environment, with females from freshwater taking an intermediate time to mature (Tukey's tests: all *p* < 0.001) (Figure 4a; Table S3). There was, however, no significant difference in size at maturity between the two salinity environments (Tukey's test: *p* = 0.202). Females from the stable‐saline environment (Tukey's test: *p* = 0.007), but not the fluctuating‐saline environment (Tukey's test: *p* = 0.326), were significantly larger than those from freshwater (Table S4). Notably, the effect of developmental environment on size at maturity (*p* = 0.009) became non‐significant after we applied a Bonferroni correction (adjusted *α* = 0.003). The developmental environment also affected adult growth, but this effect changed with age (LMM, *F* = 7.911, *p* < 0.001). Females from the fluctuating‐saline environment did not differ in size from those from the freshwater or stable‐saline environment for the first 3 weeks after maturation (Tukey's tests: all *p* > 0.068), but adult size was significantly larger in the stable‐saline than in the freshwater treatments throughout adulthood (Tukey's tests: all *p* < 0.011). Five weeks after maturation, females from the stable‐saline environment became significantly larger than those from the fluctuating‐saline environment (Tukey's tests: all *p* < 0.017; Figure 4b,d).

#### 3.2.2 Males

The developmental environment affected male age and size at maturity (LMM, both *F* > 18.961; *p* < 0.001). Unlike females, salinity fluctuations did not influence male age at maturity (Tukey's test: stable versus fluctuating, *p* = 0.168), but males from freshwater matured significantly later than those from either saline environment (Tukey's tests: both *p* < 0.001) (Figure 4a; Table S3). Males from the fluctuating‐saline environment were significantly smaller at maturity than those from the stable‐saline or freshwater environment (Tukey's tests: both *p* < 0.001), with no difference between the latter two environments (Tukey's test: *p* = 0.168; Table S4). An environment‐by‐age interaction affected male adult growth (LMM, *F* = 5.869; *p* = 0.003), but males from the fluctuating‐saline environment remained significantly smaller than those from the stable‐saline or freshwater environment throughout adulthood (Tukey's tests: all *p* < 0.001), with no difference between the latter two environments (Tukey's tests: all *p* > 0.100; Figure 4c,e).

### 3.3 Mortality and somatic maintenance

#### 3.3.1 Females

**FIGURE 5:**
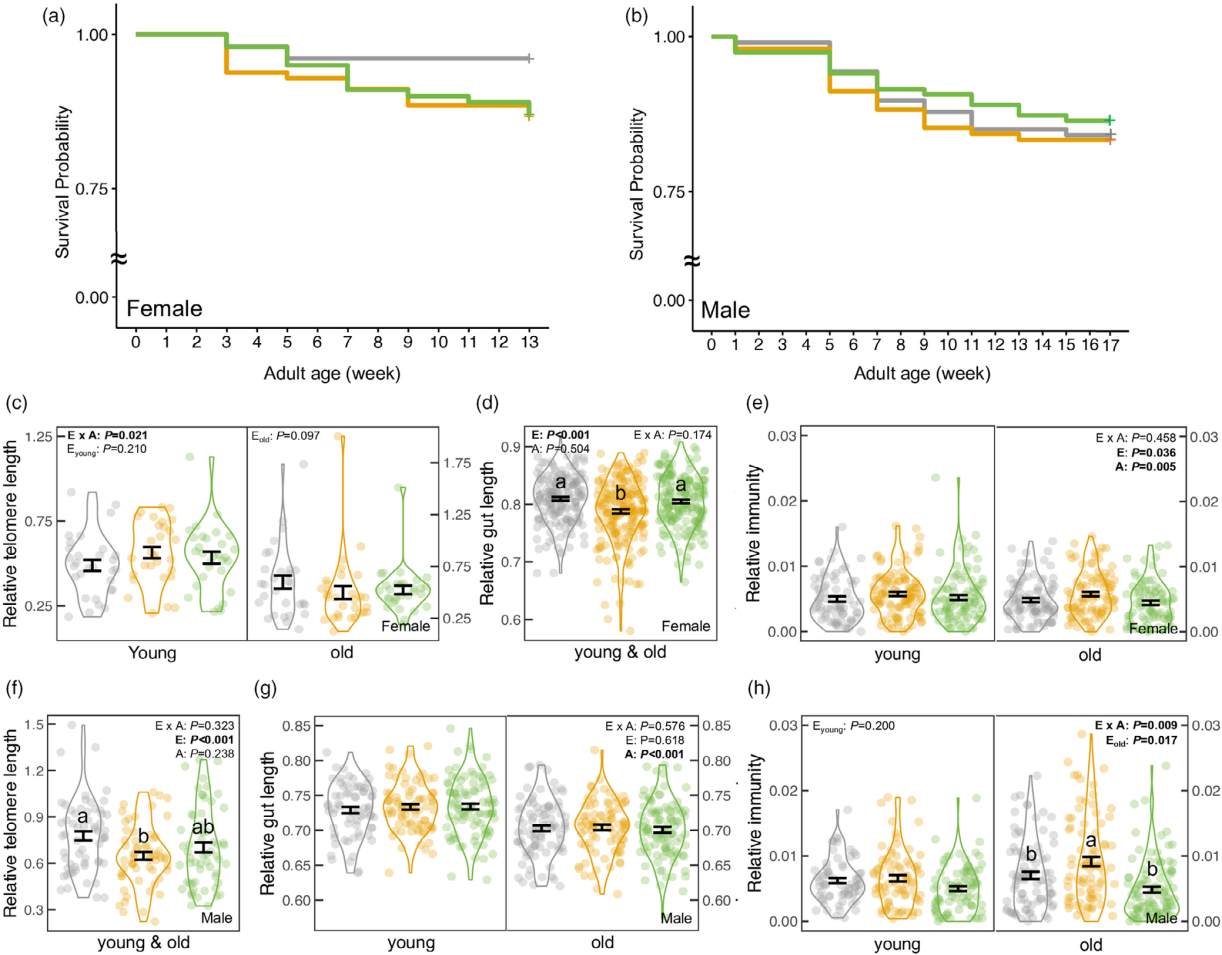
Effects of developmental environment and adult age on (a, b) mortality and self‐maintenance traits (relative telomere length, relative gut length, immunity) for (c–e) females and (f–h) males. Adult mortality was estimated from maturation to the end of the experiment. The female experiment ended after the mating period (12 weeks after 1‐week post‐maturation). The male experiment concluded after the initial measurement of mating behaviour and sperm traits (2 weeks after 1‐week post‐maturation), followed by a 12‐week mating period and repeated measurements of behaviour and sperm traits (2 weeks), totalling 17 weeks. Colours represent environments: Freshwater (grey), stable‐saline (orange), and fluctuating‐saline (green). Significance levels of environment (E), age (A) and the interaction (E × A) are shown. With a significant interaction or age effect, the environment effects are shown separately for each age class. Error bars indicate mean ± SE. Different letters represent significant differences between environments from Tukey's tests.

The developmental environment did not affect female survival (Cox, *χ* ^2^ = 5.110; *p* = 0.078; Figure 5a). There was a significant environment‐by‐age interaction on female relative telomere length (RTL) (LMM, *F* = 3.998; *p* = 0.021), but when examining young and old females separately, environment did not affect RTL (LMM, both *F* < 2.430; *p* > 0.096; Figure 5c). The interaction affecting female RTL (*p* = 0.021) became non‐significant after accounting for multiple testing (adjusted *α* = 0.003). Female RTL did not change with chronological age (LMM, *F* = 1.835; *p* = 0.178).

The developmental environment affected relative gut length (LMM, *F* = 11.874; *p* < 0.001; Figure 5d), which did not change with female age (*F* = 0.447; *p* = 0.504): females from the stable‐saline environment had a shorter gut than size‐matched females from the freshwater or fluctuating‐saline environment (Tukey's tests: both *p* < 0.001), with no difference between the latter two environments (Tukey's test: *p* = 0.855).

When female size was controlled for (LMM, *F* = 31.952; *p* < 0.001), the developmental environment affected immunity (LMM, *F* = 3.351; *p* = 0.036), irrespective of adult age (interaction: LMM, *F* = 0.781; *p* = 0.458). However, there were no significant pairwise differences between any of the environments (Tukey's tests: all *p* > 0.052; Figure 5e). Younger females had a greater immune response than older females (LMM, *F* = 8.036; *p* = 0.005). The independent effects of developmental environment (*p* = 0.036) and age (*p* = 0.005) were both non‐significant after accounting for multiple testing (Tables S1 and S5).

#### 3.3.2 Males

The developmental environment did not affect male survival (Cox, *χ* ^2^ = 0.477; *p* = 0.778; Figure 5b); however, it did affect male RTL (LMM, *F* = 7.908; *p <* 0.001), irrespective of age (*F* = 1.451; *p* = 0.238). Males from the stable‐saline environment had a shorter RTL than those from freshwater (Tukey's test: *p* < 0.001), while RTL from both environments did not differ from RTL from the fluctuating‐saline environment (Tukey's tests: both *p* > 0.064; Figure 5f). Chronological age did not affect male RTL (LMM, *F* = 0.136; *p* = 0.713).

The environment did not affect male relative gut length (neither the main effect nor its interaction with age: LMM, both *F* < 0.554; *p* > 0.575), but older males had a relatively shorter gut than younger ones (LMM, *F* = 72.657; *p* < 0.001; Figure 5g). The environment affected the immunity of older males (LMM, *F* = 4.126; *p* = 0.017) but not that of younger males (LMM, *F* = 1.622; *p* = 0.200) (interaction: LMM, *F* = 4.764; *p* = 0.009; Figure 5h). When male size was accounted for (LMM, *F* = 30.676; *p* < 0.001), older males from the stable‐saline environment exhibited a greater immune response than size‐matched males from the freshwater (Tukey's test: *p* = 0.049) or fluctuating‐saline environment (*p* = 0.027), with no difference between the latter two environments (*p* = 0.875). The interactive effect on male immunity (*p* = 0.009) was non‐significant after we applied a Bonferroni correction (adjusted *α* = 0.003) (Table S6).

### 3.4 Female fecundity

**FIGURE 6:**
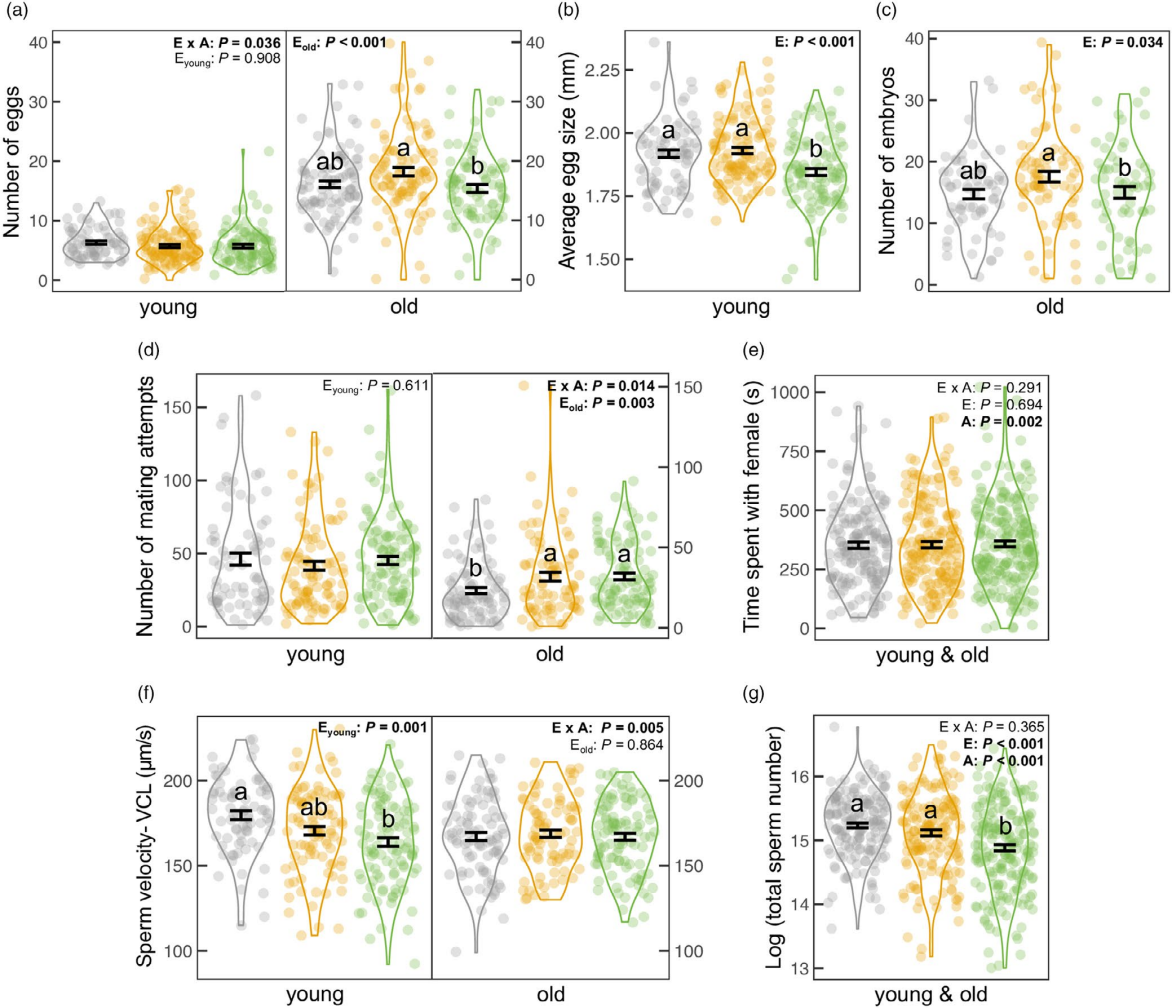
Effect of developmental environment and adult age on reproductive effort for both sexes. Colours indicate environments: Freshwater (grey); stable‐saline (orange); fluctuating‐saline (green). Female traits: (a) total egg number, (b) egg size of young females, (c) embryo number of old females; and male traits: (d) number of mating attempts, (e) time spent pursuing females, (f) sperm velocity, (g) total sperm count are presented with the significance levels of environment (E), age (A) and their interaction (E × A). Given a significant interaction or age effect, the environment effect was shown separately for each age class. Figures show mean ± SE and only indicate the conditional part of the results for (c) embryo number and (d) number of mating attempts. Different letters represent significant differences between environments based on Tukey's tests.

The developmental environment and age interacted to determine total egg number (GLMM, *χ* ^2^ = 6.642; *p* = 0.036). The environment affected the egg number of old females (LMM, *F* = 7.517; *p* < 0.001), but not that of young ones (GLMM, *χ* ^2^ = 0.193; *p* = 0.908). Old females from the fluctuating‐saline environment produced significantly fewer eggs than those from the stable‐saline environment (Tukey's test: *p* < 0.001), while neither differed from females from freshwater (Tukey's tests: both *p* > 0.078; Figure 6a). Egg size was also affected by the environment (LMM, *F* = 12.642, *p* < 0.001). The eggs of females from the fluctuating‐saline environment were smaller than those of females from the stable‐saline or freshwater environment (Tukey's tests: both *p* < 0.002), with no difference between the latter two environments (Tukey's test: *p* = 0.855; Figure 6b).

The developmental environment affected the likelihood of carrying embryos (GLMM, *χ* ^2^ = 6.145, *p* = 0.046) and, if present, the number of embryos (GLMM, *χ* ^2^ = 6.755, *p* = 0.034). The likelihood did not differ between the saline environments (Tukey's test: *p* = 0.290), but females from the stable‐saline environment carried more embryos than females from the fluctuating‐saline environment (Tukey's test: *p* = 0.042; Figure 6c). In contrast, females from the stable‐saline environment were less likely to have embryos than those from freshwater (Tukey's test: *p* = 0.037), but the number of embryos did not differ (Tukey's test: *p* = 0.161).

The likelihood of giving birth was unaffected by the environment (GLMM, *χ* ^2^ = 0.759; *p* = 0.684), but the total number of offspring differed (GLMM, *χ* ^2^ = 7.865, *p* = 0.020). Females from the stable‐saline environment produced more offspring than those from freshwater (Tukey's test: *p* = 0.047), while neither differed from females from the fluctuating‐saline environment (Tukey's tests: both *p* > 0.050).

Four of the five environment‐related effects on female fecundity—namely, *the interaction with age* on total egg number (*p* = 0.036) and *the main effects* on the likelihood of carrying embryo (*p* = 0.046), number of embryos (*p* = 0.034), and total offspring number (*p* = 0.020)—became non‐significant after controlling for multiple testing (Tables S1 and S5), whereas the environmental effect on egg size remained significant (*p* < 0.001).

### 3.5 Male mating effort

The developmental environment affected the number of mating attempts by older males (GLMM, *χ* ^2^ = 11.367, *p* = 0.003) but not by young ones (GLMM, *χ* ^2^ = 0.986, *p* = 0.611) (interaction: GLMM, *χ* ^2^ = 8.498, *p* = 0.014; Figure 6d). Old males from both saline environments (Tukey's test: stable versus fluctuating: *p* = 0.994) made more mating attempts than those from freshwater (Tukey's test: both *p* < 0.016), although the interaction between developmental environment and age (*p* = 0.014) became non‐significant after we applied a Bonferroni correction (adjusted *α* = 0.003). There was no main environmental effect on the likelihood of a successful mating attempt, or the time spent pursuing females (Figure 6e; Table S6).

### 3.6 Sperm traits

An environment‐by‐age interaction affected sperm velocity (LMM, *F* = 5.478, *p =* 0.005). The environmental effect was significant for young males (LMM, *F* = 7.014, *p =* 0.001) but not for old ones (LMM, *F* = 0.146, *p =* 0.864; Figure 6f). Sperm velocity was significantly greater for young males from the freshwater than fluctuating‐saline environment (Tukey's test: *p* < 0.001), while neither differed from that of males from the stable‐saline environment (Tukey's tests: both *p* > 0.102). However, the environment‐by‐age interaction on sperm velocity (*p =* 0.005) was non‐significant after accounting for multiple testing (adjusted *α* = 0.003).

The developmental environment and age independently affected sperm count (LMM, both *F* > 21.533, *p* < 0.001; interaction: *F* = 1.014, *p* = 0.365). Sperm count from the fluctuating‐saline environment was significantly lower than that from the stable‐saline or freshwater environments (Tukey's test: both *p* < 0.001), with no difference between the latter two environments (Tukey's test: *p* = 0.051; Figure 6g).

## 4 DISCUSSION

Fish developing in stable elevated salinity grew faster and matured earlier, and females produced more offspring but experienced faster reproductive ageing (i.e. a lower likelihood of carrying embryos when old) (Table 1). After accounting for multiple testing, the increase in female reproductive output and accelerated reproductive ageing were no longer significant; however, fish from the stable‐saline environment still showed poorer somatic maintenance (i.e. decreases in male telomere and female gut length) than those from freshwater. These results support the ‘internal PAR’ hypothesis (Figure 1), although there was no evidence of a shorter lifespan. Surprisingly, however, fluctuations in salinity induced a different life‐history response that is more aligned with the ‘silver spoon’ hypothesis (Figure 1): slower growth, lower reproductive effort in males (fewer sperm) and females (smaller eggs) and an increase in female relative gut length (Table 1). Applying Bonferroni corrections did not alter the significance of these findings. Again, there was no evidence of a shorter lifespan. Together, salinity and its fluctuations influenced growth, reproduction and self‐maintenance in different ways. We therefore reject the prediction (I) that fluctuations in salinity simply magnify life‐history changes induced by stable elevated salinity.

Effect sizes differed significantly between the sexes for some traits (Figure 2): in the fluctuating‐saline environment, female delayed maturation to reach a normal size, while males matured earlier at a smaller body size. These results partly support our prediction (II) that females ‘live slow’ in saline environments to repair salinity‐induced damage and improve their chances of adult survival (i.e. an increase in adult gut length and no decline in relative telomere length or lifespan). Our findings also partly support prediction (III) that males in saline environments accelerate the onset of reproduction to ‘live fast’, although there was no evidence of faster senescence or a shorter lifespan. Importantly, this sex difference in ‘pace of life’ only occurred when salinity fluctuated. Our study is the first to show that fluctuating versus constant salinity during development causes different life‐history responses, which are also sex‐specific.

### 4.1 Fluctuating and stable elevated salinity have different effects on growth

Mosquitofish developed faster in stable elevated salinity than in freshwater, but had slower growth when salinity fluctuated. Consequently, juveniles were about 14% smaller in size (2–3 mm) when developing in fluctuating‐ than stable‐saline environments over 6 weeks (Figure 3). This difference might reflect the greater costs required to acclimate to frequent changes in salinity (Evans & Kültz, 2020), or reflect the greater stress associated with a higher maximum salinity (20‰). Indeed, routine oxygen consumption and urea excretion rates in *Gambusia* both increased more steeply between 10‰ and 20‰ salinities than between 0‰ and 10‰ salinities (Uliano et al., 2010). This suggests an accelerating rise in energy cost with further increases in salinity. Similar to our findings, in Yarra pygmy perch, *Nannoperca obscura*, a moderate increase in salinity promoted juvenile growth, but further increases in salinity then slowed growth (Mahon et al., 2015). Studies have argued that salinity levels similar to plasma osmotic concentration (~10‰) are optimal for body functioning (Bœuf & Payan, 2001). This seems unlikely in mosquitofish though, because direct exposure of adults to saltwater can impair survivorship (Nordlie & Mirandi, 1996), alongside a significant cost of osmoregulation (Tsai et al., 2018). It is possible, however, that adults in our study handled the costs of stable elevated salinity because they acclimated to higher salinity as juveniles (Walker et al., 2020). Interestingly, Dupont‐Prinet et al. (2010) documented a trade‐off between growth and tolerance of food stress in seabass, *Dicentrarchus labrax*. They suggested that slower growth resulted from increased allocation to survival‐enhancing traits in a more challenging environment (Sokolova, 2013), which could include elevated allocation to osmoregulatory expenditures when salinity fluctuates (Evans & Kültz, 2020). Slower growth is likely to be disadvantageous in nature as juveniles are more vulnerable to predation and resource competition (Stige et al., 2019). Consequently, mosquitofish might be less invasive and weaker competitors of native fish in habitats with salinity fluctuations, such as estuaries (Alcaraz et al., 2008), because these fluctuations slow juvenile growth.

### 4.2 Stable elevated salinity accelerates reproduction, with sex‐specific changes in self‐maintenance

In our study, mosquitofish that developed in stable elevated salinity matured sooner and produced more offspring. Similar patterns arise in other fish, such as *Poecilia latipinna* (i.e. earlier maturation; Trexler & Travis, 1990) and *Rutilus caspicus* (i.e. larger gonads; Naddafi et al., 2005). In the wild, *Gambusia* in habitats with elevated salinity also have a higher gonadosomatic index and a larger brood size than those from freshwaters (Alcaraz & García‐Berthou, 2007; Brown‐Peterson & Peterson, 1990; Martin et al., 2009), while some studies reported a lower recruitment rate of *G. holbrooki* in more saline environments (Ruiz‐Navarro et al., 2011). It is, however, challenging to draw conclusions from population comparisons because (a) other factors (e.g. predation pressure, prey abundance) may covary with salinity (Gomes & Monteiro, 2007), and (b) male reproductive data are often missing from field studies (Brown‐Peterson & Peterson, 1990; Martin et al., 2009). Even so, our study indicates that stable elevated salinity during development can accelerate the onset of reproduction for both male and female mosquitofish. Notably, however, the net benefit of earlier reproduction for overall reproductive success is unclear, as the observed increase in female reproductive output because non‐significant after controlling for multiple testing.

The somatic maintenance costs of stable elevated salinity seem to vary between the sexes, as indicated by a significant difference in effect sizes (Figure 2): males experienced greater telomere shortening. Telomere shortening is widely used to indicate the ability to handle environmental challenges (Chatelain et al., 2020). Faster growth and increased osmoregulation are both associated with greater oxidative stress (Luo & Liu, 2011; Smith et al., 2016), which might accelerate telomere shortening (Armstrong & Boonekamp, 2023; Monaghan & Ozanne, 2018). But why was the effect on telomere shortening much pronounced in males? One explanation is stronger selection on females than males for longevity (Bonduriansky et al., 2008). In polyandrous species, like *G. holbrooki*, males employ intensive ‘wear‐and‐tear’ reproductive strategies once they start to breed because of intense sexual selection (Chung et al., 2024). This strategy results in more rapid senescence due to investment in reproduction over self‐maintenance. An alternative explanation is that males have a shorter lifespan than females (Pyke, 2005), making males proportionally older than females when we measured their telomere length at the same absolute age. Another possibility is that mitochondria function more efficiently in females than males due to maternal inheritance (Hill, 2019). Consequently, elevated osmoregulation might increase energy consumption and impose more oxidative damage in males. Greater oxidative damage is also expected to impair the immunity of males more than that of females (Tobler et al., 2011). Indeed, salinity‐driven differences in swellings after a PHA challenge was observed only in males but not females. However, these differences in male immunity became non‐significant after correcting for multiple testing, suggesting that salinity‐induced changes in immune response may be less pronounced than those in telomere shortening. On the contrary, the shorter guts of females in the stable‐saline environment point to a ‘hidden’ cost for females. There is a well‐documented trade‐off between gut length and other energy‐expensive tissues (Aiello & Wheeler, 1995; Kotrschal et al., 2013). It is plausible that a shorter gut is an adaptation that allows for faster growth and maturation in a stable‐saline environment (Secor, 2001).

### 4.3 Fluctuating salinity reduces reproduction effort, with sex‐specific changes in age and size at maturity

When salinity was fluctuating rather than stably elevated, both sexes performed worse in growth and reproduction; however, females developed a longer gut, with no difference in RTL in either sex (Table 1). These findings suggest that fish exposed to fluctuating salinity during development allocate more to self‐maintenance, thereby diverting resources from other key functions. Our results corroborate other fish studies where a more variable environment, albeit for temperature not salinity, slowed development (Kupren et al., 2011) and lowered fecundity (Podrabsky et al., 2008). If we assume that fluctuating environments are more stressful, this fits the general pattern that individuals that develop in stressful environments perform worse as adults (i.e. a more stable environment creates a ‘silver spoon’). The lack of a reduction in telomere length or elevation in mortality rate in our study is also consistent with a recent meta‐analysis that reported no increase in adult mortality when individuals experience a poor start in life (Cooper & Kruuk, 2018).

Females and males differed in their developmental pace when salinity fluctuated rather than remained stable: females took longer to reach maturity but matured at the same size, while males took the same time to reach maturity but were smaller (Figure 2). This sex difference aligns with the developmental threshold model (Day & Rowe, 2002). In this model, reduced growth rates lead to delayed maturation when the threshold size (i.e. the minimum sizes necessary for reproduction) is larger. Because female poeciliids typically reach a larger size than males before reproducing (Evans et al., 2011), it is plausible that female *G. holbrooki* delay maturation under unfavourable growth conditions. More generally, our results are consistent with a pattern reported in many species whereby males allocate more heavily into reaching maturity rapidly and initiating reproduction, while females allocate more to sustained growth (Janicke et al., 2016). The delayed maturation of females postpones the onset of breeding and is likely to lower absolute reproductive output in a season breeder like mosquitofish (Kahn et al., 2013). In contrast, the reproductive disadvantages of smaller male size are unclear. This is because smaller male mosquitofish are less competitive in male–male fights (Harrison et al., 2018), but they are more effective at harassing females (Pilastro et al., 1997). However, given that mating is a ‘zero‐sum game’, any potential changes in male reproductive success are only realised when smaller males from a fluctuating‐salinity environment compete with those that developed in a different environment and matured at a larger size. Such competition seems unlikely because mosquitofish often inhabit isolated ponds with homogeneous water conditions. However, competition can occur between males born at different times and developing in environments with different salinity fluctuations if short‐term climate events and/or contaminant run‐offs cause temporal shifts in water salinity (Mai et al., 2022; Szklarek et al., 2022).

## 5 CONCLUSION

We show that stable elevated salinity leads to ‘accelerated reproduction’, whereas fluctuating salinity causes ‘reduced reproductive performance’. Researchers might underestimate the impact of freshwater salinisation on reproductive output, and subsequent population dynamics if they overlook the effects of variation in salinity. We suggest that future studies should pay more attention to sex differences in the effects of climate variability. It is often stated that males are more sensitive than females to environmental changes (Iossa, 2019; Mauvais‐Jarvis, 2024). Here we show that females, notably their egg size, were strongly affected by environmental variability. Of course, care should be taken in generalising our results because salinity tolerance varies among fish species (Zhao et al., 2021). However, mosquitofish thrive in a range of polluted environments, and other freshwater species that have more restricted habitats might experience even higher reductions in reproduction when salinity fluctuates (Beatty et al., 2011).

## AUTHOR CONTRIBUTIONS

Meng‐Han Joseph Chung, Rebecca J. Fox and Michael D. Jennions conceived the study. Meng‐Han Joseph Chung, Daniel W. A. Noble, Rebecca J. Fox and Michael D. Jennions designed the experiment. Meng‐Han Joseph Chung and Lauren M. Harrison developed telomere measurement methodology. Meng‐Han Joseph Chung collected the data, performed the analyses, visualized and interpreted the results. Daniel W. A. Noble and Michael D. Jennions contributed to data analysis and interpretation. Meng‐Han Joseph Chung drafted the manuscript, with Daniel W. A. Noble and Michael D. Jennions providing critical revisions. All authors gave final approval for publication.

## FUNDING INFORMATION

Our research was supported by the ARC (DP190100279: Michael D. Jennions; DP210101152: Daniel W. A. Noble).

## CONFLICT OF INTEREST STATEMENT

The authors declare no competing interests.

## ETHICS STATEMENT

The project received approval from the Australian National University's Animal Ethics Committee (Ethics Protocol: A2021/04).

## Supporting information


**Figure S1.** Relationship between body size (log‐transformed) and gut length (log‐transformed).
**Table S1.** Summary of the main effects of developmental environment (E) and age (A), and their interaction (E × A) on each trait.
**Table S2.** Statistical outputs of environment effect on growth of (a) juveniles, (b) adult females and (c) adult males.
**Table S3.** Statistical outputs of environment effect on age at maturity of (a) females and (b) males.
**Table S4.** Statistical outputs of environment effect on size at maturity of (a) females and (b) males.
**Table S5.** Statistical outputs for the effects of developmental environment and adult age on female life‐history and reproductive traits.
**Table S6.** Statistical outputs for the effects of developmental environment and adult age on male life‐history and reproductive traits.

## Data Availability

All data and codes are available at Mendeley Data: https://data.mendeley.com/datasets/zcm8yt5cgx/1 (Chung et al., 2025).
