# Supplementary material for: Fluctuating salinity during development impacts fish life histories
Source: J Anim Ecol. 2025 Jul 1;94(9):1848–65. doi: 10.1111/1365-2656.70095 (PMC12424281; doi:10.1111/1365-2656.70095)
Supplement: Supplementary file 1 — Figure S1. Relationship between body size (log‐transformed) and gut length (log‐transformed). Table S1. Summary of the main effects of developmental environment (E) and age (A), and their interaction (E × A) on each trait. Table S2. Statistical outputs of environment effect on growth of (a) juveniles, (b) adult females and (c) adult males. Table S3. Statistical outputs of environment effect on age at maturity of (a) females and (b) males. Table S4. Statistical outputs of environment effect on size at maturity of (a) females and (b) males. Table S5. Statistical outputs for the effects of developmental environment and adult age on female life‐history and reproductive traits. Table S6. Statistical outputs for the effects of developmental environment and adult age on male life‐history and reproductive traits. [file JANE-94-1848-s001.pdf]

SUPPORTING INFORMATION

for article:

Fluctuating salinity during development impacts fish life histories

Chung, M. H. J., Noble, D. W. A., Fox, R. J., Harrison, L. M. & Jennions, M. D.

Correspondence: Meng-Han Joseph Chung (chungmenghan@gmail.com)

Methods for sperm traits

Total sperm count

After the behavioural trial, the male was anaesthetized in icy water, put on a glass slide covered with a 1% polyvinyl alcohol solution and then placed under a dissecting microscope. His gonopodium was swung forward, and we gently pressed on the abdomen to empty his sperm reserves. Males were then returned to their individual tanks for 7 days to allow for sperm replenishment (O'Dea *et al.* 2014). We examined the count and velocity of replenished sperm to standardize sperm age and to avoid any effects on sperm count of differences in copulation rates during the mating trial. After 7 days, the males were re-stripped to collect the sperm into a known volume of extender medium (207 mM NaCl, 5.4 mM KCl, 1.3 mM CaCl2, 0.49 mM MgCl2, 0.41 mM MgSO4, 10 mM Tris (Cl); pH 7.5) using a 100-µl pipette. The sperm solution was subsequently vortexed for 30 seconds and mixed using 10-ul pipette several times. We placed 3µl of the solution on a 20-micron capillary slide (Leja) to measure sperm number using the program CEROS Sperm Tracker (Hamilton Thorne Research, Beverly, MA, USA) under 100× magnification. The sperm number was analysed using the mean value of five randomly selected subsamples per male (repeatability: $r \pm SE = 0.829 \pm 0.010$, $P < 0.001$, $n = 525$ male-ages). The count data of 28 samples were not tested due to the unavailability of slides during the Covid-19 lockdown.

Sperm velocity

While stripping sperm reserves from focal males under the microscope, we collected two samples (each of 4 sperm bundles) into individual Eppendorf tubes containing 2µl of extender medium. Sperm velocity was calculated as the weighted average of the motile sperm tracks in both samples. In each Eppendorf tube, we pipetted the sperm solution and placed 3µl into the centre of a cell in a 12-cell multi-test slide (MP Biomedicals, USA) covered with 1% polyvinyl alcohol solution. We activated the sperm sample using a 3µl solution of 125 mM KCl and 2 mg/ml bovine serum albumin for 30 seconds and covered it with a coverslip. Using the CEROS Sperm Tracker (Hamilton Thorne Research), we measured (a) average path velocity (VAP): the average velocity over a smoothed cell path, (b) curvilinear velocity (VCL): the actual velocity along the trajectory, and (c) straight-line velocity (VSL). Because VAP and VSL were highly correlated with VCL (VAP-VCL: $r = 0.996$; VSL-VCL: $r = 0.998$, $n = 553$), we focused on the actual velocity of sperm cells (VCL) (Boschetto *et al.* 2011).

qPCR reaction for relative telomere length

Genomic DNA of the tailed muscles was extracted using DNeasy Blood and Tissue Kit (QIAGEN), and its concentration was quantified using Qubit™ dsDNA BR Assay Kits (Thermo Fisher Scientific). We followed the telomere primers in a previous *Gambusia* study (Rollings *et al.* 2014), and used MC1R-F (5'-CCTGTAGGCGTAGATGAGCG-3') and MC1R-R (5'-CACCAGTCCCTTCTGCAACT-3') as the control single-copy gene. qPCR reactions were run using the QuantStudio®3 system.

For each sample of telomere and MC1R, reactions were run in triplicate on 96-well plates using the QuantStudio® 3 qPCR system. Each well contained 1uL of 20ng/uL DNA sample with 9ul of master mix, including 5ul 2x SensiMix SYBR No-ROX (Meridian Bioscience Inc), 3.4ul Milli-Q water, 0.3ul of 10uM for both forward and reverse primers. For telomere amplification, an initial step started at 95°C for 10 minutes following by a total of 40 cycles of 95°C for 15 seconds, 60°C for 15 seconds and 72°C for 15 seconds. Afterwards, a melt curve stage (95°C for 15 seconds, 60°C for 60 seconds and 95°C for 15 seconds) was created to ensure qPCR specificity. For MC1R amplification, we established a 3-minute denaturation at 95°C, then a total of 40 PCR cycles at 95°C for 15 seconds, 60°C for 30 seconds and 72°C for 20 seconds, and a melt curve analysis (95°C for 15 seconds, 60°C for 60 seconds and 95°C for 15 seconds) after each run. Standard curves for both telomere and MC1R were generated using five serial dilutions of DNA (0.01, 0.1, 1, 5 and 20 ng/µL) run on each plate with an acceptance threshold (100 ± 20 %) of amplification efficiency (E), using the formula $E = 10^{(-1/\text{slope of the standard curve})}$. A negative control and two inter-plate calibrators (i.e., reference samples) were also run on each plate. RTL was assessed by the mean crossing threshold (Ct) values of each sample using the following equation (Pfaffl 2001; Monteforte *et al.* 2020):

$$RTL = \frac{(E_{Tel})^{CtTel(reference)-CtTel(sample)}}{(E_{MC1R})^{CtMC1R(reference)-CtMC1R(sample)}}$$

E is the mean amplification efficiency of either telomere or MC1R on each plate. *CtTel(reference)* and *CtMC1R(reference)* are the mean Ct values of the two calibrator DNA samples in the plate for telomere and MC1R, respectively. *CtTel(sample)* and *CtMC1R(sample)* are the average Ct values from the three repeated measurements of each sample for telomere and MC1R, respectively. In total, 29 of 2880 reactions (~1%) were excluded as undetermined data due to pipetting error. The Ct values of each sample were highly repeatable (MC1R: $r \pm$ SE = 0.980 ± 0.002, $P < 0.001$; telomere: $r \pm$ SE = 0.950 ± 0.004, $P < 0.001$, $n = 465$).

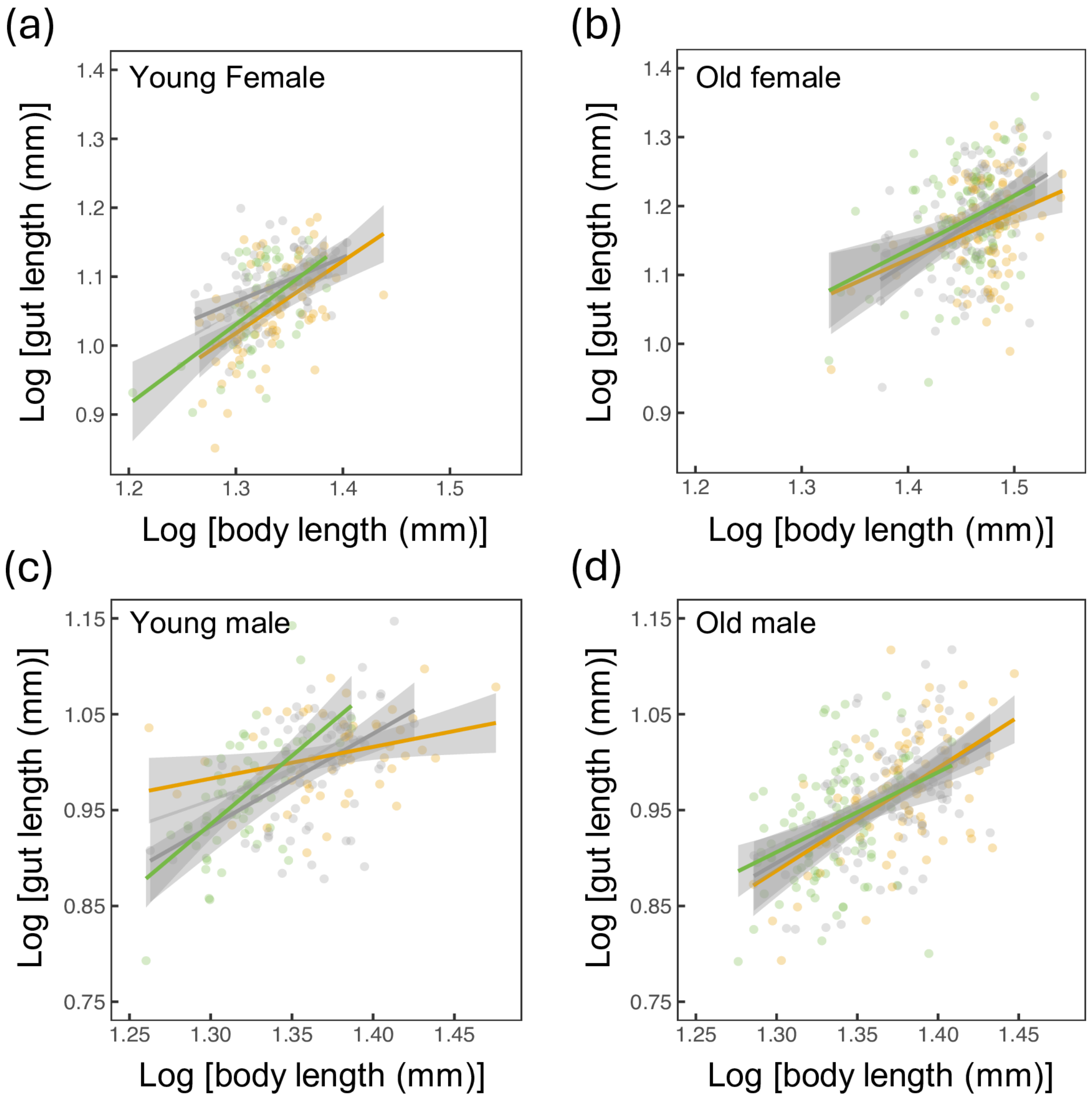

Figure S1. Relationship between body size (log-transformed) and gut length (log-transformed). (a) Young female; (b) old female; (c) young male; and (d) old male. Colours represent three developmental environments: freshwater (grey); stable salinity (orange); fluctuating salinity (green). The allometric relationships are shown along with regression lines and 95% confidence intervals. The statistical outputs are in Tables S5c and S6c.

Table S1. Summary of the main effects of developmental environment (E) and age (A), and their interaction (E×A) on each trait. "Yes" indicates a significant effect and "No" a non-significant effect, with the corresponding *P*-value. "NA" indicates no available data. The age effect (main effect or interaction) was not assessed (i.e., marked as NA) for traits measured only once (i.e., age and size at maturity, egg size of young virgin females, total embryo number, overall mortality, likelihood of giving birth, total offspring number). The main effect of environment or age was not tested (i.e., marked as NA) if there was a significant environment-by-age interaction (Engqvist 2005). Detailed statistical results are provided in the following tables and main text.

	Trait		E × A	E	A
Juvenile	Growth		**Yes (<0.001)**	NA	NA
Female	Age at maturity		NA	**Yes (<0.001)**	NA
	Size at maturity		NA	**Yes[2] (0.009)**	NA
	Relative telomere length		**Yes[1, 2] (0.021)**	NA	NA
	Relative gut length		No (0.174)	**Yes (<0.001)**	No (0.504)
	Immunity		No (0.458)	**Yes[1, 2] (0.036)**	**Yes[2] (0.005)**
	No. eggs		**Yes[2] (0.036)**	NA	NA
	Egg size		NA	**Yes (<0.001)**	NA
	No. embryos	zero-inflation	NA	**Yes[2] (0.046)**	NA
		condition	NA	**Yes[2] (0.034)**	NA
	Mortality		NA	No (0.078)	NA
	Adult growth		**Yes (<0.001)**	NA	NA
	Likelihood of giving birth		NA	No (0.684)	NA
	No. offspring	zero-inflation	NA	No (0.145)	NA
		condition	NA	**Yes[2] (0.020)**	NA
Male	Age at maturity		NA	**Yes (<0.001)**	NA
	Size at maturity		NA	**Yes (<0.001)**	NA
	Relative telomere length		No (0.323)	**Yes (<0.001)**	No (0.238)
	Relative gut length		No (0.576)	No (0.618)	**Yes (<0.001)**
	Immunity		**Yes[2] (0.009)**	NA	NA
	No. mating attempts	zero-inflation	**Yes[2] (0.011)**	NA	NA
		condition	**Yes[2] (0.014)**	NA	NA
	Likelihood of successful mating		**Yes[1] (<0.001)**	NA	NA
	Time spent with female		No (0.291)	No (0.694)	**Yes (0.002)**
	Sperm velocity		**Yes[2] (0.005)**	NA	NA
	No. sperm		No (0.365)	**Yes (<0.001)**	**Yes (<0.001)**
	Mortality		NA	No (0.788)	NA
	Adult growth		**Yes (0.003)**	NA	NA
Number of testings (N)			16	18	6
**Bonferroni-adjusted α (= 0.05/N)**			**0.0031**	**0.0027**	**0.0083**

Note [1] indicates a significant environment effect (*interaction with age*: female relative telomere length and male likelihood of successful mating; *main effect*: female immunity) with no significant differences in post hoc pairwise comparisons (see *Results* in the main text).

Note [2] indicates an effect that becomes non-significant after applying a Bonferroni correction.

Table S2. Statistical outputs of environment effect on growth of (a) juveniles, (b) adult females and (c) adult males.

(a) Juvenile growth

(i) Model with environment*age interaction

**Fixed effect**	Estimate	*SE*	*F*	*df*	*df.res*	*P*
Intercept (freshwater)	6.988	0.109	4092.564	1	151.1	**<0.001**
Environment (stable)	0.571	0.079	26.999	2	3231.5	**<0.001**
Environment (fluctuating)	0.409	0.079				
Age	1.189	0.033	1332.562	1	3186.3	**<0.001**
Age^2	1.358	0.072	359.168	1	3185.8	**<0.001**
Environment (stable) * Age	0.185	0.020	220.088	2	3187.3	**<0.001**
Environment (fluctuating) * Age	-0.230	0.020				

**Random effect**	Variance	*sd*	Number of groups
Brood ID	0.796	0.892	91
Residual	1.095	1.046	

Due to a significant environment*age interaction, environment effects were tested for separately at each age:

(ii) Juvenile age: Week 0

**Fixed effect**	Estimate	*SE*	*F*	*df*	*df.res*	*P*
Intercept (freshwater)	7.301	0.050	21407.719	1	117.030	**<0.001**
Environment (fluctuating)	-0.051	0.034	1.986	2	834.140	0.138
Environment (stable)	-0.067	0.034				

**Random effect**	Variance	*sd*	Number of groups
Brood ID	0.182	0.427	91
Residual	0.120	0.347	

(iii) Juvenile age: Week 2

**Fixed effect**	Estimate	*SE*	*F*	*df*	*df.res*	*P*
Intercept (freshwater)	11.086	0.113	9641.140	1	117.350	**<0.001**
Environment (fluctuating)	-0.035	0.074	202.730	2	775.820	**<0.001**
Environment (stable)	1.093	0.074				

**Random effect**	Variance	*sd*	Number of groups
Brood ID	0.933	0.966	91
Residual	0.520	0.721	

**Pairwise comparison**	*P*
Freshwater-Fluctuating	0.887
Freshwater-Stable	**<0.001**
Stable-Fluctuating	**<0.001**

(iv) Juvenile age: Week 4

**Fixed effect**	Estimate	*SE*	*F*	*df*	*df.res*	*P*
Intercept (freshwater)	14.605	0.143	10381.640	1	142.970	**<0.001**
Environment (fluctuating)	-0.512	0.119	251.920	2	760.040	**<0.001**
Environment (stable)	1.578	0.119				

**Random effect**	Variance	*sd*	Number of groups
Brood ID	1.259	1.122	91
Residual	1.262	1.123	

**Pairwise comparison**	*P*
Freshwater-Fluctuating	**<0.001**
Freshwater-Stable	**<0.001**
Stable-Fluctuating	**<0.001**

(v) Juvenile age: Week 6

**Fixed effect**	Estimate	*SE*	*F*	*df*	*df.res*	*P*
Intercept (freshwater)	17.459	0.156	12560.720	1	182.870	**<0.001**
Environment (fluctuating)	-1.025	0.156	176.640	2	728.630	**<0.001**
Environment (stable)	1.371	0.158				

**Random effect**	Variance	*sd*	Number of groups
Brood ID	1.118	1.057	91
Residual	2.138	1.462	

**Pairwise comparison**	*P*
Freshwater-Fluctuating	**<0.001**
Freshwater-Stable	**<0.001**
Stable-Fluctuating	**<0.001**

(b) Growth of adult females

(i) Model with environment*age interaction

**Fixed effect**	Estimate	*SE*	*F*	*df*	*df.res*	*P*
Intercept (freshwater)	17.495	0.252	4806.835	1	704.160	**<0.001**
Environment (stable)	0.620	0.235	3.548	2	369.750	**0.030**
Environment (fluctuating)	0.266	0.240				
Age	-0.264	0.035	58.207	1	1481.260	**<0.001**
Age^2	4.092	0.156	686.216	1	1483.570	**<0.001**
Environment (stable) * Age	0.033	0.015	7.911	2	1487.990	**<0.001**
Environment (fluctuating) * Age	-0.027	0.015				

**Random effect**	Variance	*sd*	Number of groups
Female ID	1.992	1.412	319
Brood ID	0.897	0.947	96
Residual	1.007	1.003	

Due to a significant environment*age interaction, environment effects were tested for separately at each age:

(ii) Female adult age: Week 1 after maturity

**Fixed effect**	Estimate	*SE*	*F*	*df*	*df.res*	*P*
Intercept (freshwater)	21.405	0.171	15560.796	1	199.800	**<0.001**
Environment (stable)	0.724	0.186	7.680	2	260.140	**<0.001**
Environment (fluctuating)	0.336	0.189				

**Random effect**	Variance	*sd*	Number of groups
Brood ID	1.113	1.055	96
Residual	1.531	1.237	

**Pairwise comparison**	*P*
Freshwater-Stable	**<0.001**
Freshwater-Fluctuating	0.182
Stable-Fluctuating	0.069

(iii) Female adult age: Week 3 after maturity

**Fixed effect**	Estimate	*SE*	*F*	*df*	*df.res*	*P*
Intercept (freshwater)	23.698	0.193	15089.085	1	202.160	**<0.001**
Environment (stable)	0.668	0.216	4.963	2	252.220	**0.008**
Environment (fluctuating)	0.262	0.219				

**Random effect**	Variance	*sd*	Number of groups
Brood ID	1.267	1.126	95
Residual	2.022	1.422	

**Pairwise comparison**	*P*
Freshwater-Stable	**0.006**
Freshwater-Fluctuating	0.457
Stable-Fluctuating	0.118

(iv) Female adult age: Week 5 after maturity

**Fixed effect**	Estimate	*SE*	*F*	*df*	*df.res*	*P*
Intercept (freshwater)	25.180	0.206	14915.214	1	219.720	**<0.001**
Environment (stable)	0.762	0.249	5.846	2	254.110	**0.003**
Environment (fluctuating)	0.092	0.254				

**Random effect**	Variance	*sd*	Number of groups
Brood ID	0.990	0.995	95
Residual	2.755	1.660	

**Pairwise comparison**	*P*
Freshwater-Stable	**0.007**
Freshwater-Fluctuating	0.931
Stable-Fluctuating	**0.016**

(v) Female adult age: Week 7 after maturity

**Fixed effect**	Estimate	*SE*	*F*	*df*	*df.res*	*P*
Intercept (freshwater)	26.674	0.221	14485.765	1	211.270	**<0.001**
Environment (stable)	0.998	0.264	9.171	2	245.840	**<0.001**
Environment (fluctuating)	0.081	0.270				

**Random effect**	Variance	*sd*	Number of groups
Brood ID	1.269	1.126	95
Residual	3.035	1.742	

**Pairwise comparison**	*P*
Freshwater-Stable	**<0.001**
Freshwater-Fluctuating	0.952
Stable-Fluctuating	**0.001**

(vi) Female adult age: Week 9 after maturity

**Fixed effect**	Estimate	*SE*	*F*	*df*	*df.res*	*P*
Intercept (freshwater)	27.464	0.230	14167.388	1	204.020	**<0.001**
Environment (stable)	0.966	0.273	10.111	2	241.230	**<0.001**
Environment (fluctuating)	-0.128	0.277				

**Random effect**	Variance	*sd*	Number of groups
Brood ID	1.510	1.229	95
Residual	3.132	1.770	

**Pairwise comparison**	*P*
Freshwater-Stable	**0.001**
Freshwater-Fluctuating	0.890
Stable-Fluctuating	**<0.001**

(vii) Female adult age: Week 13 after maturity

**Fixed effect**	Estimate	*SE*	*F*	*df*	*df.res*	*P*
Intercept (freshwater)	28.988	0.242	14250.316	1	201.090	**<0.001**
Environment (stable)	0.856	0.289	8.428	2	235.850	**<0.001**
Environment (fluctuating)	-0.261	0.297				

**Random effect**	Variance	*sd*	Number of groups
Brood ID	1.645	1.282	95
Residual	3.485	1.867	

**Pairwise comparison**	*P*
Freshwater-Stable	**0.010**
Freshwater-Fluctuating	0.656
Stable-Fluctuating	**<0.001**

(c) Growth of adult males

(i) Model with environment*age interaction

Fixed effect	Estimate	*SE*	*F*	*df*	*df.res*	*P*
Intercept (freshwater)	23.340	0.171	18553.540	1	267.660	**<0.001**
Environment (stable)	0.279	0.204	80.463	2	291.320	**<0.001**
Environment (fluctuating)	-1.952	0.200				
Age	0.095	0.009	119.042	1	1472.490	**<0.001**
Age^2	-0.291	0.041	49.798	1	1472.860	**<0.001**
Environment (stable) * Age	0.011	0.004	5.869	2	1473.570	**0.003**
Environment (fluctuating) * Age	0.013	0.004				

Random effect	Variance	*sd*	Number of groups
Male ID	1.820	1.349	327
Brood ID	0.727	0.853	98
Residual	0.097	0.311	

Due to a significant environment*age interaction, environment effects were tested for separately at each age:

(ii) Male adult age: Week 1 after maturity

Fixed effect	Estimate	*SE*	*F*	*df*	*df.res*	*P*
Intercept (freshwater)	23.293	0.179	16830.178	1	222.620	**<0.001**
Environment (stable)	0.261	0.217	76.007	2	279.750	**<0.001**
Environment (fluctuating)	-2.030	0.213				

Random effect	Variance	*sd*	Number of groups
Brood ID	0.947	0.973	98
Residual	2.094	1.447	

Pairwise comparison	*P*
Freshwater-Stable	0.455
Freshwater-Fluctuating	**<0.001**
Stable-Fluctuating	**<0.001**

(iii) Male adult age: Week 3 after maturity

Fixed effect	Estimate	*SE*	*F*	*df*	*df.res*	*P*
Intercept (freshwater)	22.906	0.168	18540.847	1	216.070	**<0.001**
Environment (stable)	0.335	0.206	76.859	2	262.370	**<0.001**
Environment (fluctuating)	-1.918	0.203				

Random effect	Variance	*sd*	Number of groups
Brood ID	0.806	0.898	97
Residual	1.841	1.357	

Pairwise comparison	*P*
Freshwater-Stable	0.240
Freshwater-Fluctuating	**<0.001**
Stable-Fluctuating	**<0.001**

(iv) Male adult age: Week 5 after maturity

Fixed effect	Estimate	*SE*	*F*	*df*	*df.res*	*P*
Intercept (freshwater)	23.088	0.171	18178.328	1	223.260	**<0.001**
Environment (stable)	0.429	0.215	73.453	2	260.800	**<0.001**
Environment (fluctuating)	-1.860	0.208				

Random effect	Variance	*sd*	Number of groups
Brood ID	0.717	0.847	96
Residual	1.939	1.392	

Pairwise comparison	*P*
Freshwater-Stable	0.118
Freshwater-Fluctuating	**<0.001**
Stable-Fluctuating	**<0.001**

(v) Male adult age: Week 7 after maturity

Fixed effect	Estimate	*SE*	*F*	*df*	*df.res*	*P*
Intercept (freshwater)	23.220	0.174	17746.717	1	220.890	**<0.001**
Environment (stable)	0.455	0.220	70.188	2	253.260	**<0.001**
Environment (fluctuating)	-1.831	0.213				

Random effect	Variance	*sd*	Number of groups
Brood ID	0.687	0.829	95
Residual	1.935	1.391	

Pairwise comparison	*P*
Freshwater-Stable	0.101
Freshwater-Fluctuating	**<0.001**
Stable-Fluctuating	**<0.001**

(vi) Male adult age: Week 9 after maturity

Fixed effect	Estimate	*SE*	*F*	*df*	*df.res*	*P*
Intercept (freshwater)	23.435	0.172	18583.097	1	218.630	**<0.001**
Environment (stable)	0.419	0.220	69.178	2	249.460	**<0.001**
Environment (fluctuating)	-1.815	0.211				

Random effect	Variance	*sd*	Number of groups
Brood ID	0.646	0.804	94
Residual	1.857	1.363	

Pairwise comparison	*P*
Freshwater-Stable	0.142
Freshwater-Fluctuating	**<0.001**
Stable-Fluctuating	**<0.001**

(vii) Male adult age: Week 15 after maturity

**Fixed effect**	Estimate	*SE*	*F*	*df*	*df.res*	*P*
Intercept (freshwater)	23.566	0.173	18430.711	1	217.890	**<0.001**
Environment (stable)	0.423	0.224	66.557	2	238.520	**<0.001**
Environment (fluctuating)	-1.803	0.214				

**Random effect**	Variance	*sd*	Number of groups
Brood ID	0.618	0.786	94
Residual	1.854	1.362	

**Pairwise comparison**	*P*
Freshwater-Stable	0.146
Freshwater-Fluctuating	**<0.001**
Stable-Fluctuating	**<0.001**

Table S3. Statistical outputs of environment effect on age at maturity of (a) females and (b) males.

(a) Females

**Fixed effect**	Estimate	*SE*	*F*	*df*	*df.res*	*P*
Intercept (freshwater)	63.586	1.233	2655.730	1	228.420	**<0.001**
Environment (stable)	-6.546	1.179	110.170	2	657.850	**<0.001**
Environment (fluctuating)	9.206	1.222				

**Random effect**	Variance	*sd*	Number of groups
Brood ID	88.390	9.402	116
Residual	130.730	11.434	

**Pairwise comparison**	*P*
Freshwater-Stable	**<0.001**
Freshwater-Fluctuating	**<0.001**
Stable-Fluctuating	**<0.001**

(b) Males

**Fixed effect**	Estimate	*SE*	*F*	*df*	*df.res*	*P*
Intercept (freshwater)	89.429	1.537	3377.121	1	251.340	**<0.001**
Environment (stable)	-7.020	1.576	18.962	2	601.220	**<0.001**
Environment (fluctuating)	-9.551	1.567				

**Random effect**	Variance	*sd*	Number of groups
Brood ID	117.100	10.820	116
Residual	206.500	14.370	

**Pairwise comparison**	*P*
Freshwater-Stable	**<0.001**
Freshwater-Fluctuating	**<0.001**
Stable-Fluctuating	0.168

Table S4. Statistical outputs of environment effect on size at maturity of (a) females and (b) males.

(a) Females

**Fixed effect**	Estimate	*SE*	*F*	*df*	*df.res*	*P*
Intercept (freshwater)	21.266	0.145	21625.717	1	234.750	**<0.001**
Environment (stable)	0.428	0.141	4.704	2	655.640	0.009*
Environment (fluctuating)	0.210	0.146				

**Random effect**	Variance	*sd*	Number of groups
Brood ID	1.156	1.075	116
Residual	1.874	1.369	

**Pairwise comparison**	*P*
Freshwater-Stable	**0.007**
Freshwater-Fluctuating	0.326
Stable-Fluctuating	0.202

*Note: The effect of developmental environment on size at maturity (P = 0.009) became non-significant after we applied a Bonferroni correction (adjusted α = 0.003).

(b) Males

**Fixed effect**	Estimate	*SE*	*F*	*df*	*df.res*	*P*
Intercept (freshwater)	23.180	0.147	24744	1	256.930	**<0.001**
Environment (stable)	0.281	0.155	174.670	2	589.110	**<0.001**
Environment (fluctuating)	-2.144	0.154				

**Random effect**	Variance	*sd*	Number of groups
Brood ID	0.997	0.999	116
Residual	2.006	1.417	

**Pairwise comparison**	*P*
Freshwater-Stable	0.168
Freshwater-Fluctuating	**<0.001**
Stable-Fluctuating	**<0.001**

Table S5. Statistical outputs for the effects of developmental environment and adult age on female life-history and reproductive traits

(a) Adult mortality of female

Fixed effect	Estimated coefficient	*SE*	Hazard ratio	χ^2	*df*	*P*
Environment (stable)	1.249	0.569	3.485	5.110	2	0.078
Environment (fluctuating)	1.182	0.578	3.262			

Random effect	Variance	*sd*
Brood ID (intercept)	0.492	0.702

(b) Relative telomere length

(i) Model with environment*age interaction

**Fixed effect**	Estimate	*SE*	*F*	*df*	*df.res*	*P*
Intercept (freshwater, old)	0.601	0.055	116.244	1	46.619	**<0.001**
Environment (stable)	-0.100	0.045	2.725	2	116.000	0.070
Environment (fluctuating)	-0.075	0.045				
Age (young)	-0.113	0.064	3.134	1	62.664	0.082
Environment (stable) * Age (young)	0.175	0.063	3.998	2	116.000	0.021*
Environment (fluctuating) * Age (young)	0.121	0.063				

**Random effect**	Variance	*sd*	correlation	Number of groups
Brood ID (intercept)	0.062	0.250		
Brood ID (young)	0.060	0.245	-0.950	35
Residual	0.030	0.174		

*Note: The interaction affecting female RTL (P = 0.021) became non-significant after accounting for multiple testing (adjusted α = 0.003).

(ii) Effect of absolute age (birth to age at sampling) on relative telomere length of females

**Fixed effect**	Estimate	*SE*	*F*	*df*	*df.res*	*P*
Intercept (freshwater, old)	0.702	0.091	56.788	1	144.100	**<0.001**
Absolute age (standardized)	-0.104	0.074	1.835	1	136.360	0.178
Environment (stable)	-0.115	0.046	3.201	2	120.690	**0.044**
Environment (fluctuating)	-0.064	0.045				
Adult age (young)	-0.322	0.163	3.694	1	156.720	0.056
Environment (stable) * Adult Age (young)	0.187	0.064	4.547	2	116.040	**0.013**
Environment (fluctuating) * Adult Age (young)	0.133	0.064				

**Random effect**	Variance	*sd*	correlation	Number of groups
Brood ID (intercept)	0.061	0.247		35
Brood ID (young)	0.062	0.249	-0.940	
Residual	0.030	0.173		

Due to a significant environment*age interaction, environment effects were tested for separately at each age:

(iii) Relative telomere length of young females

**Fixed effect**	Estimate	*SE*	*F*	*df*	*df.res*	*P*
Intercept (freshwater)	0.489	0.034	208.246	1	78.535	**<0.001**
Environment (stable)	0.075	0.042	1.602	2	58.000	0.210
Environment (fluctuating)	0.045	0.042				

**Random effect**	Variance	*sd*	Number of groups
Brood ID	0.008	0.089	30
Residual	0.026	0.163	

(iv) Relative telomere length of old females

**Fixed effect**	Estimate	*SE*	*F*	*df*	*df.res*	*P*
Intercept (freshwater)	0.599	0.056	113.429	1	47.516	**<0.001**
Environment (stable)	-0.100	0.047	2.429	2	58.000	0.097
Environment (fluctuating)	-0.075	0.047				

**Random effect**	Variance	*sd*	Number of groups
Brood ID	0.061	0.248	30
Residual	0.034	0.184	

(c) Relative gut length (log-transformed)

(i) Initial model with environment*age interaction

**Fixed effect**	Estimate	*SE*	*F*	*df*	*df.res*	*P*
Intercept (freshwater, old)	2.572	0.020	15883.71	1	352.12	**<0.001**
Environment (stable)	-0.040	0.021	3.364	2	472.40	**0.035**
Environment (fluctuating)	0.014	0.022				
Age (young)	0.052	0.034	2.296	1	455.09	0.130
Log-transformed absolute body size (standardized)	0.162	0.014	127.627	1	527.13	**<0.001**
Environment (stable) * Age (young)	-0.055	0.031	1.754	2	507.27	0.174
Environment (fluctuating) * Age (young)	-0.049	0.032				

**Random effect**	Variance	*sd*	correlation	Number of groups
Brood ID (intercept)	0.003	0.056		115
Brood ID (young)	0.007	0.085	-0.940	
Residual	0.020	0.143		

(ii) Final model excluding environment*age interaction to interpret the main effects

**Fixed effect**	Estimate	*SE*	*F*	*df*	*df.res*	*P*
Intercept (freshwater, old)	2.586	0.019	18486.128	1	334.720	**<0.001**
Environment (stable)	-0.066	0.015	11.874	2	506.270	**<0.001**
Environment (fluctuating)	-0.008	0.016				
Age (young)	0.020	0.029	0.447	1	412.720	0.504
Log-transformed absolute body size (standardized)	0.164	0.014	131.942	1	526.990	**<0.001**

**Random effect**	Variance	*sd*	correlation	Number of groups
Brood ID (intercept)	0.003	0.057		115
Brood ID (young)	0.008	0.088	-0.950	
Residual	0.020	0.143		

**Pairwise comparison**	*P*
Freshwater-Stable	**<0.001**
Freshwater-Fluctuating	0.855
Stable-Fluctuating	**<0.001**

(iii) Exclusion of the non-significant interactions did not significantly reduce model fit in the final model

	No. parameter	AIC	BIC	Log-likelihood	Deviance	χ^2_2	*P*
Initial model (i)	11	-535.75	-487.95	278.87	-557.75		
Final model (ii)	9	-536.17	-497.06	277.08	-554.17	3.577	0.167

(d) Immune response

(i) Initial model with environment*age interaction

**Fixed effect**	Estimate	*SE*	*F*	*df*	*df.res*	*P*
Intercept (freshwater, old)	0.087	0.013	40.652	1	362.370	**<0.001**
Environment (stable)	0.021	0.012	3.215	2	466.260	**0.041**
Environment (fluctuating)	-0.010	0.013				
Age (young)	0.043	0.019	4.872	1	431.750	**0.028**
Body length (standardised)	0.042	0.007	31.007	1	498.110	**<0.001**
Environment (stable) * Age (young)	-0.004	0.018	0.781	2	503.480	0.458
Environment (fluctuating) * Age (young)	0.016	0.018				

**Random effect**	Variance	*sd*	correlation	Number of groups
Brood ID (intercept)	0.001	0.024		115
Brood ID (young)	<0.001	0.011	-0.93	
Residual	0.007	0.083		

(ii) Final model excluding the non-significant interaction to interpret the main effects

**Fixed effect**	Estimate	*SE*	*F*	*df*	*df.res*	*P*
Intercept (freshwater, old)	0.085	0.013	42.230	1	358.900	**<0.001**
Environment (stable)	0.018	0.009	3.351	2	508.220	0.036*
Environment (fluctuating)	-0.002	0.009				
Age (young)	0.047	0.016	8.036	1	379.460	0.005*
Body length (standardised)	0.042	0.007	31.952	1	500.260	**<0.001**

**Random effect**	Variance	*sd*	correlation	Number of groups
Brood ID (intercept)	0.001	0.025		115
Brood ID (young)	<0.001	0.011	-0.90	
Residual	0.007	0.083		

*Note: The independent effects of developmental environment (P = 0.036) and age (P = 0.005) were both non-significant after accounting for multiple testing.

**Pairwise comparison**	*P*
Freshwater-Stable	0.100
Freshwater-Fluctuating	0.982
Stable-Fluctuating	0.053

(iii) Exclusion of the non-significant interactions did not significantly reduce model fit in the final model

	No. parameter	AIC	BIC	Log-likelihood	Deviance	χ^2_2	*P*
Initial model (i)	11	-1150.2	-1102.6	586.1	-1172.2	1.596	0.450
Final model (ii)	9	-1152.6	-1113.7	585.3	-1170.6		

(e) Total egg number

(i) Initial model with environment*age interaction

**Fixed effect**		Estimate	*SE*	χ^2	*df*	*P*
Intercept (freshwater, old)		2.764	0.041	4498.274	1	**<0.001**
Environment (stable)		0.087	0.045	16.392	2	**<0.001**
Environment (fluctuating)		-0.090	0.047			
Age (young)		-0.937	0.069	186.933	1	**<0.001**
Environment (stable) * Age (young)		-0.146	0.086	6.642	2	0.036*
Environment (fluctuating) * Age (young)		0.048	0.091			
**Random effect**	Variance	*sd*	correlation	Number of groups		
Brood ID (intercept)	0.062	0.249		115		
Brood ID (young)	0.043	0.207	-0.700			

*Note: The environment-by-age interaction on total egg number (*P* = 0.036) became non-significant after controlling for multiple testing (adjusted α = 0.003).

Due to a significant environment*age interaction, environment effects were tested for separately at each age:

(ii) Total egg number of young females

**Fixed effect**	Estimate	*SE*	χ^2	*df*	*P*
Intercept (freshwater)	1.800	0.056	1046.169	1	**<0.001**
Environment (stable)	-0.025	0.068	0.193	2	0.908
Environment (fluctuating)	-0.003	0.072			

Random effect	Variance	*sd*	Number of groups
Brood ID	0.048	0.218	110

(iii) Total egg number of old females

Fixed effect	Estimate	*SE*	*F*	*df*	*df.res*	*P*
Intercept (freshwater)	16.496	0.690	569.505	1	190.780	**<0.001**
Environment (stable)	1.701	0.783	7.517	2	229.780	**<0.001**
Environment (fluctuating)	-1.261	0.804				

Random effect	Variance	*sd*	Number of groups
Brood ID	16.470	4.058	95
Residual	24.910	4.991	

Pairwise comparison	*P*
Freshwater-Stable	0.079
Freshwater-Fluctuating	0.263
Stable-Fluctuating	**<0.001**

(f) Egg size of young females

Fixed effect	Estimate	*SE*	*F*	*df*	*df.res*	*P*
Intercept (freshwater)	1.918	0.015	15372.239	1	281.1	**<0.001**
Environment (stable)	0.010	0.019	12.642	2	258.7	**<0.001**
Environment (fluctuating)	-0.074	0.020				

Random effect	Variance	*sd*	Number of groups
Brood ID	0.001	0.035	110
Residual	0.018	0.134	

Pairwise comparison	*P*
Freshwater-Stable	0.855
Freshwater-Fluctuating	**0.001**
Stable-Fluctuating	**<0.001**

(g) Embryo number of old females

(i) Zero-inflation part of the model

**Fixed effect**	Estimate	*SE*	χ^2	*df*	*P*
Intercept (freshwater)	-0.700	0.251	7.757	1	**0.005**
Environment (stable)	-0.907	0.366	6.145	2	0.046*
Environment (fluctuating)	-0.345	0.347			

**Random effect**	Variance	*sd*	Number of groups
Brood ID	0.546	0.739	95

**Pairwise comparison**	*P*
Freshwater-Stable	**0.037**
Freshwater-Fluctuating	0.580
Stable-Fluctuating	0.290

*Note: The environment effect on the likelihood of carrying embryo ($P = 0.046$) became non-significant after controlling for multiple testing (adjusted $\alpha = 0.003$).

(ii) Conditional part of the model

**Fixed effect**	Estimate	*SE*	χ^2	*df*	*P*
Intercept (freshwater)	2.693	0.062	1894.261	1	**<0.001**
Environment (stable)	0.137	0.075	6.755	2	0.034*
Environment (fluctuating)	-0.042	0.081			

**Random effect**	Variance	*sd*	Number of groups
Brood ID	0.065	0.256	95

**Pairwise comparison**	*P*
Freshwater-Stable	0.161
Freshwater-Fluctuating	0.862
Stable-Fluctuating	**0.042**

*Note: The environment effect on the number of embryos ($P = 0.034$) became non-significant after controlling for multiple testing (adjusted $\alpha = 0.003$).

(h) Likelihood of giving birth

Fixed effect	Estimate	*SE*	χ^2	*df*	*P*
Intercept (freshwater)	-1.881	0.331	32.291	1	**<0.001**
Environment (stable)	-0.116	0.432	0.759	2	0.684
Environment (fluctuating)	-0.407	0.473			

Random effect	Variance	*sd*	Number of groups
Brood ID	0.326	0.571	95

(i) Total offspring number

(i) Zero-inflation part of the model

Fixed effect	Estimate	*SE*	χ^2	*df*	*P*
Intercept (freshwater)	-0.898	0.248	13.117	1	**<0.001**
Environment (stable)	-0.688	0.355	3.867	2	0.145
Environment (fluctuating)	-0.189	0.339			

Random effect	Variance	*sd*	Number of groups
Brood ID	0.212	0.461	95

(ii) Conditional part of the model

Fixed effect	Estimate	*SE*	χ^2	*df*	*P*
Intercept (freshwater)	2.684	0.067	1626.094	1	**<0.001**
Environment (stable)	0.198	0.083	7.865	2	0.020*
Environment (fluctuating)	-0.001	0.090			

Random effect	Variance	*sd*	Number of groups
Brood ID	0.058	0.240	95

Pairwise comparison	*P*
Freshwater-Stable	**0.047**
Freshwater-Fluctuating	0.999
Stable-Fluctuating	0.051

*Note: The environment effect on total offspring number ($P = 0.020$) became non-significant after controlling for multiple testing (adjusted $\alpha = 0.003$).

Table S6. Statistical outputs for the effects of developmental environment and adult age on male life-history and reproductive traits

(a) Adult mortality of males

Fixed effect	Estimated coefficient	*SE*	Hazard ratio	χ^2	*df*	*P*
Environment (stable)	0.068	0.343	1.070	0.477	2	0.788
Environment (fluctuating)	-0.167	0.348	0.846			

Random effect	Variance	*sd*
Brood ID (intercept)	<0.001	0.020

(b) Relative telomere length

(i) Initial model with environment*age interaction

**Fixed effect**	Estimate	*SE*	*F*	*df*	*df.res*	*P*
Intercept (freshwater, old)	0.730	0.046	251.463	1	64.831	**<0.001**
Environment (stable)	-0.086	0.046	1.998	2	116.000	0.140
Environment (fluctuating)	-0.072	0.046				
Age (young)	0.079	0.054	2.047	1	89.701	0.156
Environment (stable) * Age (young)	-0.087	0.065	1.140	2	116.000	0.323
Environment (fluctuating) * Age (young)	-0.004	0.065				

**Random effect**	Variance	*sd*	correlation	Number of groups
Brood ID (intercept)	0.032	0.180		43
Brood ID (young)	0.019	0.137	-0.870	
Residual	0.032	0.178		

(ii) Final model excluding the non-significant interaction to interpret the main effects.

**Fixed effect**	Estimate	*SE*	*F*	*df*	*df.res*	*P*
Intercept (freshwater, old)	0.745	0.042	314.073	1	47.051	**<0.001**
Environment (stable)	-0.129	0.033	7.908	2	118.000	**<0.001**
Environment (fluctuating)	-0.074	0.033				
Adult age (young)	0.049	0.039	1.451	1	29.927	0.238

**Random effect**	Variance	*sd*	correlation	Number of groups
Brood ID (intercept)	0.032	0.180		43
Brood ID (young)	0.019	0.137	-0.870	
Residual	0.032	0.178		

**Pairwise comparison**	*P*
Freshwater-Stable	**<0.001**
Freshwater-Fluctuating	0.065
Stable-Fluctuating	0.210

(iii) Exclusion of the non-significant interactions did not significantly reduce model fit in the final model

	No. parameter	AIC	BIC	Log-likelihood	Deviance	χ^2_2	*P*
Initial model (i)	10	-39.8	-7.9	29.9	-59.8	2.336	0.311
Final model (ii)	8	-41.5	-15.9	28.7	-57.5		

(iv) Effect of absolute age (birth to age at sampling) on relative telomere length of females

**Fixed effect**	Estimate	*SE*	*F*	*df*	*df.res*	*P*
Intercept (freshwater, old)	0.768	0.071	110.560	1	146.070	**<0.001**
Absolute age (standardized)	-0.021	0.055	0.136	1	161.980	0.713
Environment (stable)	-0.132	0.034	7.637	2	121.360	**<0.001**
Environment (fluctuating)	-0.078	0.035				
Adult age (young)	0.009	0.111	0.006	1	170.580	0.938

**Random effect**	Variance	*sd*	correlation	Number of groups
Brood ID (intercept)	0.031	0.177		43
Brood ID (young)	0.018	0.136	-0.870	
Residual	0.032	0.179		

(c) Relative gut length (log-transformed)

(i) Initial model with environment*age interaction

**Fixed effect**	Estimate	*SE*	*F*	*df*	*df.res*	*P*
Intercept (freshwater, old)	2.188	0.014	25305.46	1	275.31	**<0.001**
Environment (stable)	-0.003	0.018	0.029	2	435.23	0.971
Environment (fluctuating)	0.002	0.019				
Age (young)	0.082	0.019	17.923	1	323.07	**<0.001**
Log-transformed absolute body size (standardized)	0.075	0.007	124.718	1	411.49	**<0.001**
Environment (stable) * Age (young)	0.014	0.027	0.553	2	453.97	0.576
Environment (fluctuating) * Age (young)	0.027	0.026				

**Random effect**	Variance	*sd*	correlation	Number of groups
Brood ID (intercept)	0.001	0.038		114
Brood ID (young)	0.001	0.030	-0.95	
Residual	0.014	0.117		

(ii) Final model excluding the non-significant interaction to interpret the main effects

**Fixed effect**	Estimate	*SE*	*F*	*df*	*df.res*	*P*
Intercept (freshwater, old)	2.182	0.012	33992.840	1	240.370	**<0.001**
Environment (stable)	0.004	0.013	0.482	2	472.650	0.618
Environment (fluctuating)	0.014	0.015				
Adult age (young)	0.097	0.011	72.657	1	96.610	**<0.001**
Log-transformed absolute body size (standardized)	0.075	0.007	124.002	1	409.44	**<0.001**

**Random effect**	Variance	*sd*	correlation	Number of groups
Brood ID (intercept)	0.001	0.037		114
Brood ID (young)	0.001	0.031	-0.92	
Residual	0.014	0.117		

(iii) Exclusion of the non-significant interactions did not significantly reduce model fit in the final model

	No. parameter	AIC	BIC	Log-likelihood	Deviance	χ^2_2	*P*
Initial model (i)	11	-705.10	-658.37	363.55	-727.10	1.126	0.570
Final model (ii)	9	-707.98	-669.74	362.99	-725.98		

(d) Immune response

(i) Initial model with environment*age interaction

**Fixed effect**	Estimate	*SE*	*F*	*df*	*df.res*	*P*
Intercept (freshwater, old)	0.151	0.013	143.8733	1	267.600	**<0.001**
Environment (stable)	0.047	0.017	10.581	2	430.750	**<0.001**
Environment (fluctuating)	-0.029	0.017				
Age (young)	-0.016	0.017	0.797	1	308.920	0.373
Body length (standardised)	0.034	0.006	31.295	1	415.750	**<0.001**
Environment (stable) * Age (young)	-0.040	0.024	4.764	2	442.870	0.009*
Environment (fluctuating) * Age (young)	0.029	0.023				

**Random effect**	Variance	*sd*	correlation	Number of groups
Brood ID (intercept)	0.002	0.045		113
Brood ID (young)	0.001	0.037	-1.000	
Residual	0.011	0.103		

*Note: The interactive effect on male immunity ($P = 0.009$) was non-significant after we applied a Bonferroni correction (adjusted $\alpha = 0.003$).

Due to a significant environment*age interaction, environment effects were tested for separately at each age:

(ii) Young males

**Fixed effect**	Estimate	*SE*	*F*	*df*	*df.res*	*P*
Intercept (freshwater)	0.138	0.010	175.146	1	229.800	**<0.001**
Environment (stable)	0.008	0.014	1.622	2	211.720	0.200
Environment (fluctuating)	-0.019	0.016				
Body length (standardised)	0.019	0.007	8.104	1	211.010	**0.005**

**Random effect**	Variance	*sd*	Number of groups
Brood ID (intercept)	<0.001	0.009	104
Residual	0.007	0.083	

(iii) Old males

**Fixed effect**	Estimate	*SE*	*F*	*df*	*df.res*	*P*
Intercept (freshwater)	0.151	0.013	125.760	1	229.440	**<0.001**
Environment (stable)	0.045	0.019	4.126	2	266.490	**0.017**
Environment (fluctuating)	-0.010	0.020				
Body length (standardised)	0.050	0.009	30.676	1	258.11	**<0.001**

**Random effect**	Variance	*sd*	Number of groups
Brood ID (intercept)	0.001	0.023	94
Residual	0.015	0.121	

**Pairwise comparison**	*P*
Freshwater-Stable	**0.049**
Freshwater-Fluctuating	0.875
Stable-Fluctuating	**0.027**

(e) Number of mating attempts

(i) Zero-inflation part of the model with environment*age interaction

**Fixed effect**	Estimate	*SE*	χ^2	*df*	*P*
Intercept (freshwater, old)	-14.460	3.941	13.462	1	**<0.001**
Environment (stable)	1.968	4.207	0.236	2	0.889
Environment (fluctuating)	1.102	4.617			
Age (young)	0.312	2.680	0.014	1	0.907
Environment (stable) * Age (young)	-12.747	4.536	9.079	2	**0.011**
Environment (fluctuating) * Age (young)	-13.845	6.601			

**Random effect**	Variance	*sd*	Number of groups
Brood ID (intercept)	<0.001	<0.001	98
Male ID (intercept)	1733.000	41.630	323

(ii) Conditional part of the model with environment*age interaction

**Fixed effect**	Estimate	*SE*	χ^2	*df*	*P*
Intercept (freshwater, old)	3.097	0.082	1440.625	1	**<0.001**
Environment (stable)	0.330	0.113	12.932	2	**0.002**
Environment (fluctuating)	0.353	0.107			
Age (young)	0.698	0.113	38.366	1	**<0.001**
Environment (stable) * Age (young)	-0.423	0.157	8.498	2	0.014*
Environment (fluctuating) * Age (young)	-0.359	0.150			

**Random effect**	Variance	*sd*	Number of groups
Brood ID (intercept)	0.032	0.180	98
Male ID (intercept)	<0.001	0.001	323

*Note: The environment-by-age interaction ($P = 0.014$) became non-significant after we applied a Bonferroni correction (adjusted $\alpha = 0.003$).

Due to a significant environment*age interaction, environment effects were tested for separately at each age:

(iii) Zero-inflation part of the model testing for the environment effect (young male)

**Fixed effect**	Estimate	*SE*	χ^2	*df*	*P*
Intercept (freshwater)	-3.255	0.610	28.458	1	**<0.001**
Environment (stable)	0.073	0.811	1.619	2	0.445
Environment (fluctuating)	-1.652	1.411			

**Random effect**	Variance	*sd*	Number of groups
Brood ID (intercept)	<0.001	<0.001	98

(iv) Conditional part of the model testing for the environment effect (young male)

**Fixed effect**	Estimate	*SE*	χ^2	*df*	*P*
Intercept (freshwater)	3.814	0.088	1885.711	1	**<0.001**
Environment (stable)	-0.100	0.113	0.986	2	0.611
Environment (fluctuating)	-0.014	0.109			

**Random effect**	Variance	*sd*	Number of groups
Brood ID (intercept)	0.015	0.124	98

(v) Zero-inflation part of the model testing for the environment effect (old male)

**Fixed effect**	Estimate	*SE*	χ^2	*df*	*P*
Intercept (freshwater)	-5.652	3.282	2.965	1	0.085
Environment (stable)	2.550	3.322	0.878	2	0.645
Environment (fluctuating)	2.003	3.340			

(vi) Conditional part of the model testing for the environment effect (old male)

**Fixed effect**	Estimate	*SE*	χ^2	*df*	*P*
Intercept (freshwater)	3.117	0.086	1316.515	1	**<0.001**
Environment (stable)	0.330	0.117	11.367	2	**0.003**
Environment (fluctuating)	0.341	0.112			

**Random effect**	Variance	*sd*	Number of groups
Brood ID (intercept)	0.014	0.118	94

**Pairwise comparison**	*P*
Freshwater-Stable	**0.015**
Freshwater-Fluctuating	**0.007**
Stable-Fluctuating	0.994

(f) Likelihood of successful mating

(i) Initial model with environment*age interaction

**Fixed effect**	Estimate	*SE*	χ^2	*df*	*P*
Intercept (freshwater, old)	-24.563	4.743	26.819	1	**<0.001**
Environment (stable)	-19.524	5.343	19.212	2	**<0.001**
Environment (fluctuating)	-0.125	3.419			
Age (young)	0.025	2.970	<0.001	1	0.993
Environment (stable) * Age (young)	22.715	4.072	39.260	2	**<0.001**
Environment (fluctuating) * Age (young)	2.600	3.595			

**Random effect**	Variance	*sd*	correlation	Number of groups
Brood ID (intercept)	1107.000	33.270		98
Brood ID (young)	4426.000	66.530	-1.000	
Male ID (intercept)	3240.000	56.920		323

Due to a significant environment*age interaction, environment effects were tested for separately at each age:

(ii) Young males: Likelihood of successful mating

**Fixed effect**	Estimate	*SE*	χ^2	*df*	*P*
Intercept (freshwater)	-1.133	0.264	18.452	1	**<0.001**
Environment (stable)	-0.022	0.356	0.040	2	0.980
Environment (fluctuating)	-0.067	0.349			

**Random effect**	Variance	*sd*	Number of groups
Brood ID (intercept)	<0.001	<0.001	98

(iii) Old males: Likelihood of successful mating

**Fixed effect**	Estimate	*SE*	χ^2	*df*	*P*
Intercept (freshwater)	-2.009	0.439	20.903	1	**<0.001**
Environment (stable)	-0.959	0.549	3.061	2	0.216
Environment (fluctuating)	-0.374	0.462			

**Random effect**	Variance	*sd*	Number of groups
Brood ID (intercept)	1.095	1.047	94

(g) Time spent with female

(i) Initial model with environment*age interaction

**Fixed effect**	Estimate	*SE*	*F*	*df*	*df.res*	*P*
Intercept (freshwater, old)	353.330	20.000	308.969	1	264.410	**<0.001**
Environment (stable)	32.310	26.950	1.393	2	461.260	0.250
Environment (fluctuating)	42.330	25.790				
Age (young)	-20.650	25.800	0.633	1	275.380	0.427
Environment (stable) * Age (young)	-35.810	33.830	1.241	2	255.000	0.291
Environment (fluctuating) * Age (young)	-51.370	32.640				

**Random effect**	Variance	*sd*	correlation	Number of groups
Brood ID (intercept)	5276	72.640		98
Brood ID (young)	7433	86.210	-0.970	
Male ID (intercept)	6942	83.320		323
Residual	21686	147.260		

(ii) Final model excluding the non-significant interaction to interpret the main effects

**Fixed effect**	Estimate	*SE*	*F*	*df*	*df.res*	*P*
Intercept (freshwater, old)	368.540	17.210	453.844	1	218.619	**<0.001**
Environment (stable)	14.020	20.290	0.367	2	287.991	0.694
Environment (fluctuating)	15.910	19.620				
Adult age (young)	-51.390	15.730	10.559	1	84.508	**0.002**

**Random effect**	Variance	*sd*	correlation	Number of groups
Brood ID (intercept)	4812	69.370		98
Brood ID (young)	7083	84.160	-0.960	
Male ID (intercept)	7112	84.330		323
Residual	21634	147.080		

(iii) Exclusion of the non-significant interactions did not significantly reduce model fit in the final model

	No. parameter	AIC	BIC	Log-likelihood	Deviance	χ^2_2	*P*
Initial model (i)	11	7372.7	7420.3	-3675.4	7350.7	2.463	0.292
Final model (ii)	9	7371.2	7410.1	-3676.6	7353.2		

(h) Sperm velocity- VCL

(i) Model with environment*age interaction

Fixed effect	Estimate	*SE*	*F*	*df*	*df.res*	*P*
Intercept (freshwater, old)	166.466	2.369	4882.279	1	265.200	**<0.001**
Environment (stable)	2.603	3.222	0.331	2	443.230	0.719
Environment (fluctuating)	1.700	3.084				
Age (young)	12.198	3.301	13.507	1	281.290	**<0.001**
Environment (stable) * Age (young)	-9.648	4.378	5.478	2	256.720	0.005*
Environment (fluctuating) * Age (young)	-13.996	4.235				

Random effect	Variance	*sd*	correlation	Number of groups
Brood ID (intercept)	63.690	7.981		97
Brood ID (young)	99.450	9.972	-0.110	
Male ID (intercept)	35.450	5.954		317
Residual	379.680	19.485		

*Note: The environment-by-age interaction on sperm velocity (*P* = 0.005) was non-significant after accounting for multiple testing (adjusted α = 0.003).

Due to a significant environment*age interaction, environment effects were tested for separately at each age:

(ii) Sperm velocity of young males

Fixed effect	Estimate	*SE*	*F*	*df*	*df.res*	*P*
Intercept (freshwater)	178.592	2.775	4126.773	1	251.530	**<0.001**
Environment (stable)	-7.043	3.428	7.014	2	224.100	**0.001**
Environment (fluctuating)	-12.614	3.361				

Random effect	Variance	*sd*	Number of groups
Brood ID (intercept)	135.000	11.620	97
Residual	451.000	21.240	

Pairwise comparison	*P*
Freshwater-Stable	0.103
Freshwater-Fluctuating	**<0.001**
Stable-Fluctuating	0.173

(iii) Sperm velocity of old males

**Fixed effect**	Estimate	*SE*	*F*	*df*	*df.res*	*P*
Intercept (freshwater)	167.038	2.323	5134.543	1	225.820	**<0.001**
Environment (stable)	1.698	3.129	0.146	2	246.040	0.864
Environment (fluctuating)	0.808	2.994				

**Random effect**	Variance	*sd*	Number of groups
Brood ID (intercept)	72.220	8.498	94
Residual	381.180	19.524	

(i) Total sperm count (log-transformed)

(i) Initial model with environment*age interaction

**Fixed effect**	Estimate	*SE*	*F*	*df*	*df.res*	*P*
Intercept (freshwater, old)	15.386	0.066	53427.953	1	234.570	**<0.001**
Environment (stable)	-0.077	0.083	8.715	2	418.540	**<0.001**
Environment (fluctuating)	-0.312	0.080				
Age (young)	-0.266	0.092	8.294	1	237.140	**0.004**
Environment (stable) * Age (young)	-0.141	0.114	1.014	2	236.030	0.365
Environment (fluctuating) * Age (young)	-0.143	0.110				

**Random effect**	Variance	*sd*	correlation	Number of groups
Brood ID (intercept)	0.109	0.331		97
Brood ID (young)	0.205	0.453	-0.910	
Male ID (intercept)	0.036	0.190		312
Residual	0.217	0.465		

(ii) Final model excluding the non-significant interaction to interpret the main effects

**Fixed effect**	Estimate	*SE*	*F*	*df*	*df.res*	*P*
Intercept (freshwater, old)	15.430	0.059	67599.516	1	181.690	**<0.001**
Environment (stable)	-0.146	0.062	21.534	2	262.170	**<0.001**
Environment (fluctuating)	-0.383	0.059				
Adult age (young)	-0.357	0.066	29.055	1	90.276	**<0.001**

**Random effect**	Variance	*sd*	correlation	Number of groups
Brood ID (intercept)	0.119	0.345		97
Brood ID (young)	0.235	0.485	-0.920	
Male ID (intercept)	0.041	0.203		312
Residual	0.208	0.456		

**Pairwise comparison**	*P*
Freshwater-Stable	0.051
Freshwater-Fluctuating	**<0.001**
Stable-Fluctuating	**<0.001**

(iii) Exclusion of the non-significant interactions did not significantly reduce model fit in the final model

	No. parameter	AIC	BIC	Log-likelihood	Deviance	χ^2_2	*P*
Initial model (i)	11	887.1	934.0	-432.6	865.1	1.841	0.398
Final model (ii)	9	885.0	923.3	-433.5	867.0		

References

Boschetto, C., Gasparini, C. & Pilastro, A. (2011). Sperm number and velocity affect sperm competition success in the guppy (*Poecilia reticulata*). *Behavioral Ecology and Sociobiology,* 65(4)**,** 813–821. https://doi.org/10.1007/s00265-010-1085-y

Engqvist, L. (2005). The mistreatment of covariate interaction terms in linear model analyses of behavioural and evolutionary ecology studies. *Animal Behaviour,* 70**,** 967–971. https://doi.org/10.1016/j.anbehav.2005.01.016

Monteforte, S., Cattelan, S., Morosinotto, C., Pilastro, A. & Grapputo, A. (2020). Maternal predator-exposure affects offspring size at birth but not telomere length in a live-bearing fish. *Ecology and Evolution,* 10(4)**,** 2030–2039. https://doi.org/10.1002/ece3.6035

O'Dea, R.E., Jennions, M.D. & Head, M.L. (2014). Male body size and condition affects sperm number and production rates in mosquitofish, *Gambusia holbrooki*. *Journal of Evolutionary Biology,* 27(12)**,** 2739–2744. https://doi.org/10.1111/jeb.12534

Pfaffl, M.W. (2001). A new mathematical model for relative quantification in real-time RT-PCR. *Nucleic Acids Research,* 29(9)**,** e45. https://doi.org/10.1093/nar/29.9.e45

Rollings, N., Miller, E. & Olsson, M. (2014). Telomeric attrition with age and temperature in Eastern mosquitofish (*Gambusia holbrooki*). *Naturwissenschaften,* 101(3)**,** 241–244. https://doi.org/10.1007/s00114-014-1142-x
